# SUMOylation modulates the LIN28A‐let‐7 signaling pathway in response to cellular stresses in cancer cells

**DOI:** 10.1002/1878-0261.12694

**Published:** 2020-06-01

**Authors:** Jinzhuo Dou, Hailong Zhang, Ran Chen, Zimei Shu, Haihua Yuan, Xian Zhao, Yanli Wang, Jian Huang, Aiwu Zhou, Jianxiu Yu

**Affiliations:** ^1^ Department of Biochemistry and Molecular Cell Biology State Key Laboratory of Oncogenes and Related Genes Shanghai Key Laboratory of Tumor Microenvironment and Inflammation Shanghai Jiao Tong University School of Medicine Shanghai China; ^2^ Department of Pathophysiology Key Laboratory of Cell Differentiation and Apoptosis of Chinese Ministry of Education Shanghai Jiao Tong University School of Medicine Shanghai China; ^3^ Department of Oncology Shanghai Ninth People’s Hospital Shanghai Jiao Tong University School of Medicine Shanghai China; ^4^ Basic Clinical Research Center Renji Hospital School of Medicine Shanghai Jiao Tong University Shanghai China

**Keywords:** binding affinity to precursor let‐7, cancer progression, let‐7 biogenesis, LIN28A, stresses, SUMOylation

## Abstract

LIN28A is a conserved RNA‐binding protein that inhibits the biogenesis of let‐7 microRNAs, thus promoting cancer progression. However, mechanisms underlying the activation of the LIN28A‐let‐7 signaling pathway remain poorly understood. Here, we show that LIN28A is SUMOylated *in* *vivo* and *in vitro* at K15, which is increased by hypoxia but reduced by chemotherapy drugs such as Cisplatin and Paclitaxel. SUMOylation of LIN28A aggravates its inhibition of let‐7 maturation, resulting in a stark reduction in let‐7, which promotes cancer cell proliferation, migration, invasion, and tumor growth *in* *vivo*. Mechanistically, SUMOylation of LIN28A increases its binding affinity with the precursor let‐7 (pre‐let‐7), which subsequently enhances LIN28A‐mediated recruitment of terminal uridylyltransferase TUT4 and simultaneously blocks DICER processing of pre‐let‐7, thereby reducing mature let‐7 production. These effects are abolished in SUMOylation‐deficient mutant LIN28A‐K15R. In summary, these findings shed light on a novel mechanism by which SUMOylation could regulate the LIN28A‐let‐7 pathway in response to cellular stress in cancer cells.

## Graphical abstract

This study demonstrated that SUMOylation of LIN28A increases the binding affinity of LIN28A with pre‐let‐7, subsequently to promote TUT4‐mediated uridylation and block DICER processing of pre‐let‐7, thus leading to the reduction of mature let‐7. SUMOylation of Lin28A is regulated in response to cancer cellular stresses such as hypoxia and chemotherapy drug treatment, which have implications in cancer prognosis and therapy.

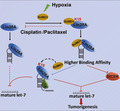

**Abbreviations**
CCK8: Cell Counting Kit‐8
CSD: cold‐shock domain
EMSA: electrophoretic mobility shift assay
miRNAs: MicroRNAs
PTM: post‐translational modification
qRT–PCR: quantitative real‐time PCR
RIP: RNA immunoprecipitation
SENPs: SUMO‐specific proteases
SPR: surface plasmon resonance
SUMO: small ubiquitin‐like modifier
TUTase: uridylyltransferase
ZKD: zinc finger domains

## 1 Introduction

MicroRNAs (miRNAs) are a class of ~ 22 nucleotide (nt), small single RNAs that participate in regulating all cellular processes. Let‐7 is the second known miRNA that is originally discovered in *Caenorhabditis elegans* (Reinhart *et al*., 2000) and subsequently found in all vertebrates (Roush and Slack, 2008). Let‐7 is highly conserved across animal species in sequence and function, and human let‐7 family comprises ten mature let‐7 family members that are processed from 13 precursor sequences (Hertel *et al*., 2012; Roush and Slack, 2008). All members of the let‐7 family contain the identical seed sequence to recognize their target genes that control cell‐fate decisions, including oncogenes *K‐RAS* (Johnson *et al*., 2005), *HMGA* (Lee and Dutta, 2007; Mayr *et al*., 2007), *c‐Myc* (Sampson *et al*., 2007), and a set of genes that regulate pluripotency maintenance (Bussing *et al*., 2008; Lin *et al*., 2007). Accumulating evidence suggests that let‐7 family members (let‐7s) function as important tumor suppressors in multiple cancers; however, let‐7s are down‐regulated in a variety of cancers and associated with increased proliferation and invasion of cancer cells (Johnson *et al*., 2007; Lu *et al*., 2005; Ricarte‐Filho *et al*., 2009; Takamizawa *et al*., 2004). The low levels of let‐7s in cancers are usually not due to both primary and precursor let‐7 (pri‐let‐7 and pre‐let‐7) levels that are comparable in most of cells. Up till now the main molecular mechanism is that the levels of mature let‐7s are post‐transcriptionally controlled by an RNA‐binding protein (RBP) LIN28, which directly binds to the terminal loop (TL) of pri‐let‐7 and pre‐let‐7, thus suppressing its biogenesis (Heo *et al*., 2008; Newman *et al*., 2008; Rybak *et al*., 2008; Viswanathan and Daley, 2010).

LIN28 was firstly discovered in the *C. elegans* during mutagenesis screenings as a heterochronic gene that regulates developmental timing (Ambros and Horvitz, 1984; Moss *et al*., 1997). In humans, there are two Lin28 family members, LIN28A, and its paralog Lin28B genes encoding 209‐ and 250‐amino acid proteins, respectively. They share overall 77% identity at protein level and contain conserved RNA‐binding domains including a cold‐shock domain (CSD) and two Cys‐Cys‐His‐Cys (CCHC) type zinc finger domains (ZKD) (Guo *et al*., 2006; Huang, 2012). The ZKD of LIN28A can attach to the motif GGAG in the TL of pre‐let‐7 adjacent to the DICER cleavage site (Nam *et al*., 2011; Rybak *et al*., 2008; Wang *et al*., 2017), while the CSD interacts with a subset of pre‐let‐7 harboring (U)GAU in the loop region (Ustianenko *et al*., 2018). Upon binding to pre‐let‐7, LIN28A recruits the terminal uridylyltransferases (TUTase) TUT4/TUT7 which catalyze oligouridylation of pre‐let‐7 at the 3′ overhang. Oligouridylated pre‐let‐7 is resistant to DICER cleavage but undergoes degradation by the DIS3L2 exonuclease (Chang *et al*., 2013; Hagan *et al*., 2009; Heo *et al*., 2008; Heo *et al*., 2009; Thornton *et al*., 2012; Ustianenko *et al*., 2013). It is worthy of attention that LIN28A has been identified to be re‐activated in ~ 15% of human cancers and regarded as a biomarker of multiple advanced cancers (Viswanathan *et al*., 2009). High level of LIN28A protein and subsequent blockage of let‐7 biogenesis are associated with tumorigenesis, invasiveness, and poor prognosis of malignancies in such as lung cancer, liver cancer, breast cancer, gastric cancer, and prostate cancer (Balzeau *et al*., 2017; Thornton and Gregory, 2012; Viswanathan *et al*., 2009). However, mechanisms underlying the LIN28A‐let‐7 signaling pathway to be activated in response to cellular stresses such as hypoxia remain poorly revealed.

SUMOylation is a reversible and dynamic post‐translational modification (PTM) that small ubiquitin‐like modifier (SUMO) conjugates to protein substrates at the lysine (K) residues by an enzymatic cascade. It is a critical PTM that is implicated in protein functions by altering stability, localization, activity, and protein–protein interaction of substrates. SUMOylation is an important mechanism in regulating cellular responses to such as genotoxic and oxidative stresses by the activation of many signaling pathways (Seeler and Dejean, 2017). Given that cancer cells are subject to stresses which appears to up‐regulate SUMOylation, we speculate SUMOylation is involved in the LIN28A‐let‐7 signaling pathway in cancer cells in response to stress stimuli.

Here, we found that LIN28A was modified by SUMO1 at K15, which was induced by the microenvironmental hypoxia, whereas repressed by genotoxic chemotherapy drugs including Cisplatin and Paclitaxel (PTX). SUMOylation of LIN28A significantly increased the binding affinity to pre‐let‐7s, subsequently promoted oligouridylation of pre‐let‐7 by TUT4 and sequestered the processing of pre‐let‐7 by DICER, thereby inhibiting let‐7 biogenesis in response to stresses such as hypoxia. Moreover, we observed an important role of LIN28A SUMOylation in promoting cancer progression.

## 2 Materials and methods

### 2.1 Antibodies and reagents

Mouse‐anti‐Flag M2 (#F1804) and mouse‐anti‐HA (MMS‐101 P) were purchased from Sigma (St. Louis, MO, USA). Rabbit‐anti‐LIN28A (#ab46020), rabbit‐anti‐SENP1 (#ab108981), and rabbit‐anti‐SUMO1 (#ab32058) were purchased from Abcam (Cambridge, UK). Mouse‐anti‐LIN28A (MA1‐016) was purchased from Thermo Fisher (Waltham, MA, USA). Mouse‐anti‐GST (66001‐1‐Ig), mouse‐anti‐His (66005‐1‐Ig), and mouse‐anti‐Alpha‐Tubulin (66031‐1‐Ig) were purchased from ProteinTech Group (Rosemont, IL, USA). Rabbit‐anti‐UBC9 (sc‐10759) was purchased from Santa Cruz Biotechnology (Dallas, TX, USA). Hydrogen peroxide solution (H_2_O_2_, #H1009), polybrene (hexadimethrine bromide, #H9268), puromycin (#P8833), and 3× Flag Peptide (F4799) were purchased from Sigma. Ni^2+^‐NTA agarose beads were purchased from Qiagen (Hilden, Germany). Protein G Plus/Protein Agarose suspension (#IP05) was purchased from Calbiochem (San Diego, CA, USA). Glutathione Sepharose 4B (#17‐0756‐01) was purchased from GE Healthcare Life Sciences (Marlborough, MA, USA).

### 2.2 Plasmids

The GV227‐LIN28A plasmid containing full‐length human LIN28A cDNA was purchased from GenePharma (Shanghai, China) and then subcloned in the pCMV‐HA, pCMV‐Myc, and N‐terminal p3 × FLAG‐CMV vectors, respectively. The LIN28A cDNA was cloned into the pGEX‐4T‐1 vector for prokaryotic expression. For stable expression, the HA‐LIN28A was cloned into the Lentiviral vector pCD513B. The LIN28A mutants and truncations were generated using the KOD‐plus‐mutagenesis Kit (TOYOBO, New York, NY, USA), as per the manufacturer’s protocol. For protein expression and purification, LIN28A‐∆14 was cloned into pET‐28a vector, and SUMO1 (amino acids 1‐96) was amplified from His‐SUMO1, and then inserted into pET‐28a‐LIN28A‐∆14 to encode SUMO1‐LIN28A‐∆14 fusion protein where residues 1‐96 of SUMO1 were fused with 15‐209 of LIN28A. The Flag‐HA‐DICER expression plasmid was kindly provided by V. Narry Kim at the Seoul National University. The Flag‐SENP expression plasmids were kindly provided by J.K. Cheng at the Shanghai Jiao Tong University School of Medicine. The shRNA oligonucleotides for LIN28A, SENP1, and UBC9 referred from Sigma were subcloned into the lentiviral vector pLKO.1. Pre‐let‐7s were cloned into the pGreen‐puro vector. The sequences of all plasmids were verified by sequencing. Primer sequences used for plasmid construction are listed in Table S1.

### 2.3 RNA labeling

The biotin‐pre‐let‐7s were transcribed and labeled using the ScriptMAX™ Thermo T7 Transcription Kit (TSK‐101; TOYOBO) and Biotin‐16‐UTP (#11388908910; Roche, Indianapolis, IN, USA) according to the manufacturer’s guidelines. PCRs were performed with the Template DNA, and primers are listed in Table S2.

### 2.4 Cell cultures

Human embryonic kidney (HEK)‐293T, 293FT, HeLa, and human prostate cancer cell line DU145 were cultured in Dulbecco’s modified Eagle’s medium (Corning, New York, NY, USA) containing 10% FBS (Biowest, Kansas, MO, USA), 1% penicillin, and streptomycin (Invitrogen, Carlsbad, CA, USA). Human breast cancer cell line T47D and SK‐BR‐3 were cultured in RPMI 1640 (HyClone, Logan, UT, USA) containing 10% FBS (Biowest), 1% penicillin, and streptomycin (Invitrogen). Above cell lines were cultured in 5% CO_2_ humidified incubator at 37 °C. Human breast cancer cell line MDA‐MB‐231 was cultured in L‐15 LEIBOVITZ (Hyclone) containing 10% FBS (Biowest), 1% penicillin, and streptomycin (Invitrogen) at 37 °C in incubator without CO_2_. Cell transfection was performed by using Lipofectamine 2000 (Invitrogen).

### 2.5 SUMOylation assays

Three methods were used to detect LIN28A SUMOylation. (a) SUMOylation analysis by Ni^2+^‐NTA pull down, LIN28A SUMOylation was analyzed *in* *vivo* by transfecting indicated plasmids into HEK‐293T cells through the method of Ni^2+^ NTA pull down as described previously (Yu *et al*., 2009); (b) SUMOylation analysis in *Escherichia coli* system, LIN28A SUMOylation was analyzed *in vitro* by co‐transforming GST‐LIN28A‐WT/K15R with pE1E2S1 into *E. coli BL21*; then, cells were harvested and lysed as previously described (Chen *et al*., 2015); (c) endogenous SUMOylated‐LIN28A was detected by co‐immunoprecipitation as previously described (Chen *et al*., 2015). In brief, T47D cells, SK‐BR‐3 cells, or DU145 over‐expressing LIN28A stable cells were lysed in ice‐cold NEM‐RIPA buffer (50 mm Tris/HCl pH 7.4, 150 mm NaCl, 1% NP‐40, 20 mm N‐ethylmaleimide, and a Complete Protease Inhibitor cocktail) for 1hr and then sonicated on ice; after centrifuging, the total cell lysates were incubated protein A/G agarose beads with anti‐IgG or anti‐LIN28A antibody overnight at 4 °C. Then, the beads were washed for three times with NEM‐RIPA buffer and followed by immunoblotting with anti‐SUMO1 and anti‐LIN28A. To identify SUMOylation of LIN28A in tissues, tissues were lysed in NEM‐RIPA buffer containing 0.1% SDS and 5 mm EDTA as previously described (Yu *et al*., 2009) and then immunoprecipitated with anti‐IgG or anti‐LIN28A antibody.

### 2.6 CCK8 assay

The stable cell line proliferation ability was detected by using the Cell Counting Kit (**#**40203ES60, TEASEN, Shanghai, China) according to the manufacturer’s guidelines.

### 2.7 Migration assay by wound healingyi

Migration mediated by LIN28A was determined by wound‐healing assay (Haq *et al*., 2019). Serum‐starved DU145 or T47D stable cell lines were cultured on 12‐well plates overnight for adhering. The cell monolayer was scraped into a certain array with sterile pipette tips and washed with PBS. Then, cells were incubated with serum‐free medium and cultured in 5% CO_2_ humidified incubator at 37 °C. Wound closure was captured at the indicated time using Nikon (Melville, NY, USA) microscope. The areas of wounds were measured by imagej software (NIH, Bethesda, MD, USA) from three independent experiments. Migration rates were represented by area ratio.

### 2.8 Three‐dimensional (3D) cell culture growth assay

The 3D cell culture was carried out according to the methods described before (Yuan *et al*., 2017; Zhao *et al*., 2017). Briefly, mixtures of 5 μL of cell suspension (1 × 10^3^ cells) and 5 μL of 3D matrix™ gel (Millipore, Darmstadt, Germany) were added to the inner well of μ‐Slides (IBIDI). The cells were covered with complete medium and cultured in 5% CO_2_ humidified incubator at 37 °C for 7 days. Pictures were captured using Nikon microscope. The number of colonies with diffuse tumorsphere was counted, and the ratio of the former to the number of total colonies was presented by histogram.

### 2.9 Northern blotting analysis

The northern blotting analysis of RNA was conducted as described before (Zhang *et al*., 2019). Briefly, total RNAs were isolated with TRIzol reagent (Invitrogen) and denatured at 95 °C for 5 min. Then, the RNAs were separated by electrophoresis on the 20% polyacrylamide 8 m urea gel and transferred to the nylon membrane (Roche). After cross‐linking, RNAs were detected with the specified probes. Biotin or DIG end‐labeled DNA oligonucleotide probes for miRNA detection were listed in Table S3.

### 2.10 RNA immunoprecipitation assay (RIP)

The RIP assay was conducted as previously described (Yuan *et al*., 2017; Zhu *et al*., 2015). Briefly, 48 h after transfection with indicated plasmids, cells were lysed in RIP lysis buffer (50 mm Tris/HCl pH 7.4, 150 mm NaCl, 2 mm MgCl_2_, 1% NP‐40, 1 mm DTT, 40U RNase inhibitor, 400 μm VRC (New England Biolabs, Ipswich, MA, USA), and Protease Inhibitor Cocktail) for 1 h on ice. Then, one‐tenth of the lysate was transferred to a new tube as input, and the others were incubated with specified antibodies and protein A/G agarose beads at 4 °C. The beads were washed with RIP lysis buffer for five times. Input RNA and RIP RNA were extracted with TRIzol reagent and then reversely transcribed by using the PrimeScript RT‐PCR Kit (#RR037A; TAKARA, Otsu, Shiga, Japan). The RNAs bound to protein were detected by real‐time PCR with SYBR Green PCR Master Mix (#4309155; Applied Biosystems, Waltham, MA, USA). The fold changes in pre‐let‐7s associate with LIN28A were first normalized by input of pre‐let‐7s and then presented as relative binding fold. Primers used in quantitative real‐time PCR (qRT–PCR) were listed in Table S4.

### 2.11 RNA pull‐down assay

293T cells transfected with HA‐LIN28A or HA‐LIN28A‐K15R were lysed in lysis buffer (25 mm Tris/HCl pH 8.0, 150 mm NaCl, 2 mm MgCl_2_, 0.5% NP‐40, 1 mm DTT, Protease Inhibitor Cocktail, and 40U RNase inhibitor) for 1 h followed by sonication on ice and centrifugation for 30 min at 4 °C. Meanwhile, 250 pol of biotin‐pre‐let‐7g was incubated with 50 μL of Dynabeads MyOne Streptavidin C1 (#65001; Invitrogen) for 30 min, and then, the beads washed with washing buffer (5 mm Tris/HCl pH 7.5, 0.5 mm EDTA, 1 m NaCl) for three times were mixed with the 293T cell lysate and incubated overnight at 4 °C. Fractions unbound to the beads were obtained from supernatant. The Dynabeads were washed with lysis buffer for three times, followed by immunoblotting with anti‐HA.

### 2.12 Protein purification

Two protein purification methods were used in our experiments.

1. pET‐28a‐SUMO1‐LIN28A‐∆14 was transformed into *E. coli BL21*, single colony was used to inoculate LB medium, and protein expression was induced with 0.25 mm IPTG. Cells were collected, resuspended, and disrupted by sonication in lysis buffer (20 mm Tris/HCl pH 7.4, 1.5 m NaCl, 5 mm DTT and 5% Glycerin), and the cell lysates were applied onto a HisTrap Ni‐FF column after centrifuged at 20 000 ***g*** for 30 min at 4 °C. The target protein was then eluted from the column with a gradient of 20–200 mm imidazole. The fractions containing SUMO1‐LIN28A‐∆14 were pooled based upon SDS/PAGE analysis and then dialyzed and loaded onto Superdex‐75 High load for further purification. SUMO1‐LIN28A‐∆14 was collected after elute with elution buffer (10 mm Tris/HCl pH 7.4, 0.15 m NaCl, 5% Glycerin). For the preparation of recombinant LIN28A‐∆14, the SUMO1‐LIN28A‐∆14 fusion protein was digested by Senp2 protease to remove the SUMO1 tag before gel filtration.
2. We conducted two sets of transfection protocols. In the first group, Flag‐LIN28A was transfected alone or together with His‐SUMO1 and HA‐UBC9 into HEK‐293T cells. In another group, Flag‐LIN28A‐WT or Flag‐LIN28A‐K15R was transfected into 293T SENP1^−/−^ cells. 48 h after transfection, cells were harvested and lysed in RIPA lysis buffer (50 mm Tris/HCl pH 7.4, 150 mm NaCl, 1% NP‐40, and one Complete Protease Inhibitor Cocktail) for 1 h on ice. The lysates were centrifugated for 30 min at 4 °C after sonication, and then, the supernatants were transferred into new tubes and incubated with anti‐Flag M2 affinity beads overnight at 4 °C. The beads were washed three times by RIPA lysis buffer, and Flag‐tagged LIN28A variants were purified using the 3× Flag peptide according to the manufacturer’s specifications (Sigma).

### 2.13 Electrophoretic mobility shift assay (EMSA)

PreE‐let‐7a‐1 and preE‐let‐7g were synthesized by GenePharma and biotin labeled at 5′‐end. Purified r.LIN28A‐∆14 or r.SUMO1‐LIN28A‐∆14 was incubated with 5 nm preE‐let‐7 probes in 20 µL of total volume‐binding buffer containing 20 mm Tris/HCl pH 7.6, 5 mm MgCl_2_, 100 mm NaCl, 10% Glycerol, 2 mm DTT, and 40U RNase inhibitor (Thermo). The reactions were incubated for 60 min at 25 °C and separated on native 7% polyacrylamide gels. The dissociation constant *K*_d_ was calculated as previously described (Piskounova *et al*., 2011). In brief, band intensities were quantified using imagej software and used to calculate fraction bound by r.LIN28A. The data were fitted to nonlinear regression (curve fit) method of graphpad prism7.0 (GraphPad Software, La Jolla, CA, USA). Dissociation constant *K*_d_ was derived from a fit to the equation: Fraction bound = *B*_max_ ([r.LIN28A])/(*K*_d_ + [r.LIN28A]), where *B*_max_ represents the observed maximum fraction of probe bound, [rLIN28A] represents protein molar concentration.

### 2.14 Surface plasmon resonance (SPR) analysis

To assess the binding affinity between LIN28A‐∆14 or SUMO1‐LIN28A‐∆14 and pre‐let‐7g, SPR by Biacore T200 instrument (GE Healthcare) was used as previously described (Jian *et al*., 2019) Briefly, 20 nm biotinylated pre‐let‐7g was captured on the surface of SA chip at a flow rate of 30 μL·min^−1^ in PBS with 0.05% (v/v) Tween‐20 and 5% DMSO, pH 7.4. Series concentrations of LIN28A‐∆14 and SUMO1‐LIN28A‐∆14 were injected into the flow system and analyzed, respectively. All binding analysis was performed in PBS with 0.05% (v/v) Tween‐20 and 5% DMSO, pH 7.4, at 25 °C. The association time and the dissociation time were set to 120 and 360 s, respectively. After dissociation, the chip surface was regenerated by 50 mm NaOH and 1 m NaCl. Prior to analysis, double reference subtractions were made to eliminate bulk refractive index changes, injection noise, and data drift. The binding affinity was determined by global fitting to a Langmuir 1 : 1 binding model within the biacore evaluation software (GE Healthcare).

### 2.15 Molecular models of SUMO1‐LIN28A‐pre‐let‐7g complex

The crystal structures of SUMO1 (PDB: 4WJQ) and LIN28A‐Let7g (PDB: 3TS2) were blindly docked at ClusPro 2.0 docking server with a distance restrain of 20 Angstroms between Ca atoms of Gly96 of Sumo1 and Gln36 of LIN28A. The top solution from the server was selected for presentations here. The electrostatic surface of SUMO1 was calculated with software APBS, and the cartoon of the structures was generated using software pymol (Schrödinger, New York, NY, USA) (Vajda *et al*., 2017).

### 2.16 *In vitro* uridylation assay

*In vitro* uridylation assay was conducted according to previously published method (Heo *et al*., 2008) with minor change. HEK‐293T cells transfected with or without Flag‐TUT4 were harvested and lysed in lysis buffer (50 mm Tris/HCl pH 7.4, 150 mm NaCl, 1% NP‐40, protein inhibitor cocktail, and 40U RNase inhibitor) on ice for 1h and then centrifugated for 30 min at 4 °C after sonication. The supernatant was transferred to new tubes and incubated with 20 µL protein A/G agarose beads and 2 µg of anti‐Flag antibody overnight at 4 °C. The protein A/G agarose coupled with TUT4 was washed with lysis buffer for three times and then used for *in vitro* uridylation reaction in a total volume of 30 µL containing 3.2 mm MgCl_2_, 1 mm DTT, 0.25 mm rNTPs (TOYOBO), and 0.5–1 µm biotin‐labeled pre‐miRNA. After incubation for 30 min at 37 °C, the RNA was isolated from the reaction mixture with TRIzol reagent (Invitrogen) and analyzed on 20% urea polyacrylamide gel.

### 2.17 *In vitro* pre‐let‐7s processing assay

HEK‐293T cells transfected with or without Flag‐HA‐DICER were harvested and lysed in lysis buffer (50 mm Tris/HCl pH 7.4, 150 mm NaCl, 1% NP‐40, protein inhibitor cocktail, and 40U RNase inhibitor) on ice for 1 h and then centrifugated for 30 min at 4 °C after sonication. The supernatant was incubated with 20 µL of protein A/G agarose beads and 2 µg of anti‐Flag antibody overnight at 4 °C, and then, the beads coupled with DICER were washed three times with lysis buffer. The DICER cleavage reactions were made according to the published protocol (Park *et al*., 2011) with minor changes. Briefly, the reaction was performed in a total volume of 40 µL containing 10 mm Tris/HCl pH 8.0, 2 mm MgCl_2_, 100 mm KCl, 0.1 mm EDTA, 1 mm DTT, 40U RNase inhibitor, 0.5–1 µm biotin‐labeled pre‐miRNA, and the protein A/G agarose beads coupled with DICER, and the reaction mixture was incubated at 37 °C for 60 min. The RNA was purified from the reaction mixture and analyzed on 20% urea polyacrylamide gel.

### 2.18 Soft agar colony‐formation assay

The soft agar colony**‐**forming assay was performed as previously described (Huang *et al*., 2012; Zhu *et al*., 2015). In brief, stable cells were suspended in 2 mL of complete cell culture medium at specified density along with 0.35% low melting Bacto agar (Amresco, Wayne, PA, USA) and seeded on six‐well plates coated with 2 mL of solidified gel containing 10% FBS and cell culture medium with 0.6% Bacto agar (Amresco). Then, cells were cultured at 37 °C in a humidified atmosphere containing 5% CO_2_ for 21 days. The viable colonies were stained with 0.005% crystal violet. The photographs were taken, and the number of colonies was counted using photoshop cc 2019 (version 20.0.0; Adobe, San Jose, CA, USA). The experiments were performed at least three independent times with triplicate repeats.

### 2.19 Xenografted tumor models

Mouse xenografted tumor models were established as previously described (Huang *et al*., 2012; Yuan *et al*., 2017). Each DU145 stable cell lines (2.5 × 10^6^) were individually injected subcutaneously into 5‐week‐old nude mice (*n* = 5) on the bilateral back. Tumors were measured every 3 days after 2 weeks of injection. All mice were euthanized 4 weeks later, and tumors were dissected for weight measurement. All animal studies were conducted under the approval and guidance of the Animal Ethics Committee of Shanghai Jiao Tong University of Medicine.

### 2.20 Cell viability assay

T47D stable cells were seeded at a density of 3 × 10^4^ cells per well in 96‐well plates. After 24 h of culture, cells were treated with or without cisplatin for 48 h. Cell viability was measured using a Cell Counting Kit‐8 (CCK8) assay Cell Counting Kit (#40203ES60; TEASEN) according to the manufacturer’s protocol. Cell viability (%) = (absorbance of treated group − blank control)/(absorbance of untreated group − blank control) × 100%.

### 2.21 Statistical analysis

Data were presented as means ± SD or ± SEM for qPCR, CCK assay, and soft agar colony‐formation assay and mouse xenograft tumor model. Statistical significance was analyzed with *t*‐test (two‐tailed and unpaired) or two‐way ANOVA by using microsoft excel and graphpad prism 7.0 (GraphPad Software). A value of *P* < 0.05 (*), <0.01 (**), or < 0.001 (***) was considered statistically significant.

## 3 Results

### 3.1 LIN28A is SUMOylated *in vitro* and *in vivo*

**Fig. 1:**
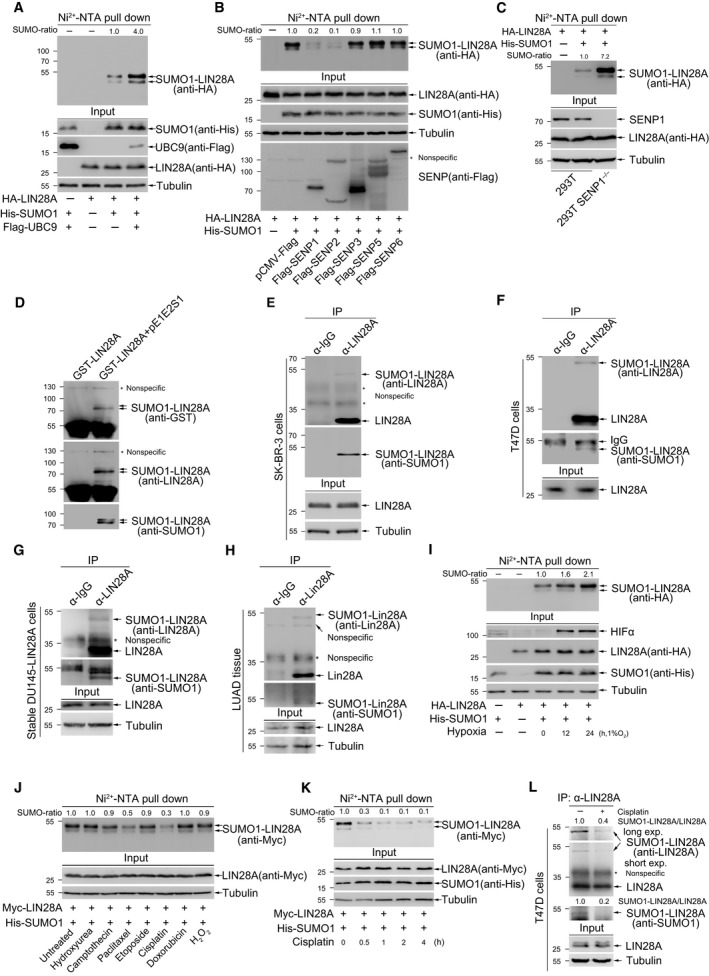
LIN28A is SUMOylated *in* *vivo* and *in vitro*. (A) LIN28A is modified by SUMO1. HA‐LIN28A with or without His‐SUMO1 and Flag‐UBC9 were co‐transfected into 293T cells. 48 h later, cells were lysed for precipitation with Ni^2+^‐NTA resin. Western blot analysis was performed with the indicated antibodies. The SUMO1‐LIN28A bands by Ni^2+^‐NTA pull down were quantified by imagej software. (B‐C) SENP1 removes the SUMOylation of LIN28A. (B) HA‐LIN28A and His‐SUMO1 were transfected with Flag‐SENPs into 293T cells. (C) 293T cells and 293T *SENP1* ^−/−^ cells were transfected with plasmids encoding HA‐LIN28A alone or together with His‐SUMO1 for 48 h. Cells were lysed for precipitation with Ni^2+^‐NTA resin. Western blot analysis was performed with the indicated antibodies. The SUMO1‐LIN28A bands were quantified by imagej software. (D) LIN28A is SUMOylated *in vitro*. Plasmid pGEX‐4T1‐LIN28A with or without pE1E2S1 plasmid was co‐transformed into *E. coli BL21*. Western blot analysis was conducted with anti‐SUMO1 antibody after GST‐pull down, and the same membrane was also detected with anti‐LIN28A and anti‐GST antibodies after stripping. (E, F) SUMOylation of endogenous LIN28A occurs naturally in SK‐BR‐3 cells (E) and T47D cells (F). SK‐BR‐3 cells or T47D cells were lysed for IP with anti‐LIN28A antibody or normal IgG, followed by western blotting with anti‐SUMO1 and anti‐LIN28A antibodies. (G) SUMOylation of LIN28A is detected in DU145 stably expressing LIN28A cells. DU145‐LIN28A cells were lysed for IP and western blot analysis as performed before. (H) SUMOylation of endogenous LIN28A occurs naturally in human LUAD tissue. LUAD tissue was lysed for IP and western blot analysis as performed before. (I) Hypoxia upregulates SUMOylation of LIN28A. 293T cells transfected with HA‐LIN28A and His‐SUMO1 were cultured in 1% of oxygen condition (hypoxia) for indicated time before being harvested. Ni^2+^‐NTA resin pull down was performed to detect SUMOylated LIN28A. Samples were immunoblotted using the indicated antibodies. The SUMO1‐LIN28A bands were quantified by imagej software. (J) Chemotherapy drugs suppress SUMOylation of LIN28A. 293T cells transfected Myc‐LIN28A and His‐SUMO1 were treated with Hydroxyurea (3 mm), Camptothecin (10 µm), PTX (30 µm), Etoposide (10 µm), Cisplatin (10 µm), and Doxorubicin (1 µm) for 6 h before being harvested. Ni^2+^‐NTA resin pull down was performed to detect SUMOylated LIN28A. Western blot was performed with the indicated antibodies. The SUMO1‐LIN28A bands were quantified by imagej software. (K) Cisplatin down‐regulates SUMOylation of LIN28A. 293T transfected Myc‐LIN28A and His‐SUMO1 plasmids were treated with Cisplatin (10 µm) for indicated time. Cells were lysed for precipitation with Ni^2+^‐NTA resin. Western blot analysis was performed with the indicated antibodies. The SUMO1‐LIN28A bands were quantified by imagej software. (L) Cisplatin reduces endogenous LIN28A SUMOylation. T47D cells treated with Cisplatin (10 µm) for 12 h were lysed for IP with anti‐LIN28A antibody, followed by western blotting with anti‐SUMO1 and anti‐LIN28A antibodies. The SUMO1‐LIN28A bands were quantified by imagej software.

To identify whether LIN28A is SUMOylated, HA‐LIN28A with His‐tagged SUMO1, SUMO2, or SUMO3 was transfected into HEK 293T cells. Then, Ni^2+^‐NTA resin precipitation assay was performed to pull**‐**down His‐SUMO1/2/3‐conjugated LIN28A. The result showed that LIN28A was SUMOylated with His‐SUMO1/2/3, and the SUMO1 modification of LIN28A was the most active among these three modifications (Fig. S1). Thus, we focused on SUMO1 modification of LIN28A in the following studies. LIN28A and His‐SUMO1 with or without SUMO‐conjugating enzyme E2 Flag‐UBC9 were transfected into HEK‐293T cells. The result showed that SUMO1 modification of LIN28A was significantly increased by UBC9 (Fig. 1A). SUMO‐specific proteases (SENPs) are cysteine proteases that can de‐SUMOylate of substrates through hydrolase or isopeptidase activity, we wondered whether SENPs can remove the LIN28A SUMO1 modification. LIN28A SUMOylation was greatly reduced by co‐transfection with SENP1 or SENP2 but not with SENP3, SENP5 or SENP6 (Fig. 1B), which was consistent with that SENP1 and SENP2 have broad specificity for SUMO1/2/3, all other SENP isoforms (SENP3, SENP5, SENP6, and SENP7) prefer SUMO2/3 over SUMO1 for deconjugation of SUMO (Kumar and Zhang, 2015). Moreover, we confirmed that SUMOylation of LIN28A was enhanced when endogenous SENP1 was knocked out in HEK‐293T cells by the CRISPR‐Cas9 system (Fig. 1C). Next to examine whether LIN28A is SUMOylated *in vitro*, we performed a prokaryotic SUMOylation assay in *E. coli BL21* co‐expressing GST‐LIN28A with the plasmid pE1E2S1, in which two enzymes E1, E2 and SUMO1 are simultaneously expressed. After GST‐pull down, immunoblotting with anti‐SUMO1 antibody showed that GST‐LIN28A co‐transformed with pE1E2S1 was SUMOylated. The SUMOylated bands were also confirmed by the detection with anti‐LIN28A and anti‐GST antibodies on the same membrane after stripping (Fig. 1D). We then examined whether endogenous LIN28A is endogenously modified by SUMO1. Previous studies have shown that LIN28A is expressed in HER2+ breast cancer cell lines such as T47D and SK‐BR‐3, and barely expressed in prostate cancer cell line DU145 (Fig. S2A,B) (Albino *et al*., 2016; Piskounova *et al*., 2011). Thus, these cell lines were used in the SUMOylation analyses with the method of immunoprecipitation (IP). SK‐BR‐3 cells, T47D cells, or DU145‐LIN28A stably expressing LIN28A were harvested in the RIPA lysis buffer, followed by IP with anti‐LIN28A antibody or normal IgG. Western blotting with anti‐SUMO1 and anti‐LIN28A antibodies showed that LIN28A was moderately modified by endogenous SUMO1 in SK‐BR‐3 (Fig. 1E), T47D (Fig. 1F), and stable DU145‐LIN28A cells (Fig. 1G). We also detected the SUMO1 modification of LIN28A in tumor tissue of lung adenocarcinoma (LUAD). The result demonstrated that the expression level of LIN28A in tumor tissue was much higher than that of in adjacent normal tissues (Fig. S2C), and a SUMOylated LIN28A band was clearly detected in lung adenocarcinoma tumor tissue (Fig. 1H). Collectively, these results demonstrate that LIN28A is SUMOylated *in vitro* and *in* *vivo*.

### 3.2 SUMOylation of LIN28A is enhanced by hypoxia while repressed by chemotherapy drugs

We wondered whether SUMOylation of LIN28A naturally occurs and can be induced or repressed by some stresses. Firstly, 293T cells were co‐transfected HA‐LIN28A and His‐SUMO1 for 24 h, and then treated with hypoxia (1% O_2_) for 0, 12, and 24 h. The result of SUMOylation assay showed that SUMOylation of LIN28A was significantly increased by hypoxia treatment in a time‐dependent manner (Fig. 1I). Considering the role of LIN28A as an oncogene, we examined effects of several chemotherapy drugs on SUMOylation of LIN28A. 293T cells were transfected with Myc‐LIN28A and His‐SUMO1 plasmids, and treated with Hydroxyurea, Camptothecin, PTX, Etoposide, Cisplatin, or Doxorubicin for 6 h before cells were harvested for SUMOylation analysis. The result showed that PTX and Cisplatin greatly reduced the SUMOylation levels of LIN28A, whereas others had no effects (Fig. 1J). To further confirm the inhibition of LIN28A SUMOylation by PTX and Cisplatin, Myc‐LIN28A or HA‐LIN28A was transfected with His‐SUMO1 into 293T cells, and then, cells were treated with Cisplatin (10 µm) or PTX (30 µm) for indicated times before the SUMOylation assay by the method of Ni^2+^‐NTA resin precipitation. The results showed that SUMOylation of LIN28A was down‐regulated after treatment with Cisplatin (Fig. 1K) and PTX (Fig. S3A) in a time‐dependent manner. Moreover, we treated T47D cells with Cisplatin (10 µm) for 6 or 12 h, and then conducted a SUMOylation analysis by the IP method, showing that the SUMOylation of endogenous LIN28A was repressed by Cisplatin (Figs 1L and S3B). T47D cells treated with PTX also showed a decrease in SUMO1 modification of LIN28A (Fig. S3C). Thus, above results reveal that SUMOylation of LIN28A can be induced by hypoxia while repressed by chemotherapy drugs such as Cisplatin and PTX.

### 3.3 LIN28A is majorly SUMOylated at K15

**Fig. 2:**
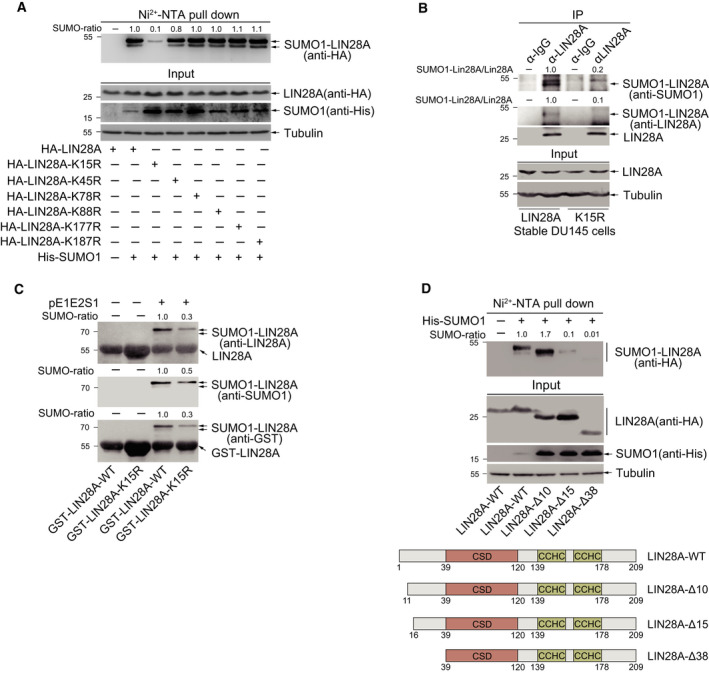
LIN28A is mainly SUMOylated at K15. (A) Mutation of K15R decreases SUMOylation of LIN28A. HA‐LIN28A‐WT or different HA‐LIN28A mutations and His‐SUMO1 were co‐transfected into 293T cells. Cells were lysed for Ni^2+^‐NTA pull down, followed by western blotting by indicated antibodies. The SUMO1‐LIN28A bands were quantified by imagej software. (B) SUMOylation at K15 of LIN28A is verified in stable DU145 cell lines by the IP method. DU145 cells stably expressing HA‐LIN28A‐WT or HA‐LIN28A‐K15R were lysed for IP with anti‐LIN28A antibody or normal IgG, followed by western blotting with anti‐SUMO1 and anti‐LIN28A antibodies. The SUMO1‐LIN28A and LIN28A (IP panels) bands were quantified by imagej software. The ratio of SUMO1‐LIN28A/LIN28A presents the intensity of SUMOylated LIN28A. (C) Mutation of K15R reduces LIN28A SUMOylation in an *E. coli* SUMOylation system. Plasmid pGEX‐4T1‐LIN28A‐WT or pGEX‐4T1‐LIN28A‐K15R with pE1E2S1 plasmid was co‐transformed into *E. coli BL21*. Western blot analysis was conducted with anti‐SUMO1 antibody after GST‐pull down, and the same membrane after stripping was also detected with anti‐LIN28A and anti‐GST antibodies. The SUMO1‐LIN28A bands were quantified by imagej software. (D) Truncated forms show K15 is a major SUMO site of LIN28A. HA‐LIN28A‐WT or different HA‐LIN28A‐truncated and His‐SUMO1 were co‐transfected into 293T cells. Cells were lysed for Ni^2+^‐NTA pull down, followed by western blotting with indicated antibodies. The SUMO1‐LIN28A bands were quantified by imagej software.

Next, we sought to identify the true SUMO sites at LIN28A protein. Since the amino acid sequence of LIN28A does not comprise the conserved SUMO‐motif ΨKXD/E, we took a strategy to mutate all 17 lysines (Ks) of LIN28A into arginine (R). In addition to point mutations, we also mutated some of them together with adjacent ones. All these mutants of LIN28A were individually transfected with His‐SUMO1 into 293T cells, and the SUMOylation assays by the method of Ni^2+^‐NTA resin precipitation revealed that the mutation of K15R greatly reduced LIN28A SUMOylation (Figs 2A and S4). To determine whether K15R is the main SUMO site of LIN28A, DU145 cells stably expressing HA‐LIN28A‐WT or K15R were harvested for SUMOylation analysis by the IP method, and the result showed that SUMOylation of LIN28A‐K15R was greatly reduced compared to that of LIN28A‐WT (Fig. 2B). We also employed the *E. coli*‐based SUMOylation assay *in vitro* and observed a decrease in SUMOylation of GST‐LIN28A‐K15R (Fig. 2C). To further confirm SUMOylation of LIN28A at K15 in cells, we generated three N‐terminal truncated constructs, LIN28A‐∆10 (aa 11–209), LIN28A‐∆15 (aa 16–209), and LIN28A‐∆38 (aa 39–209), as shown in Fig. 2D. The SUMOylation assays showed that SUMOylation of LIN28A‐∆38 and LIN28A‐∆15 but not of LIN28A‐∆10 (still containing K15 residue) was enormously decreased when compared to that of LIN28A‐WT (Fig. 2D). However, the elimination of K15 residue did not completely remove the SUMO1 modification of LIN28A, indicating the existence of other artificial modification sites in the over‐expression system. Thus, we conclude that K15 may be a major site for SUMOylation of LIN28A.

### 3.4 Mutation K15R of LIN28A weakens its oncogenic capacity

**Fig. 3:**
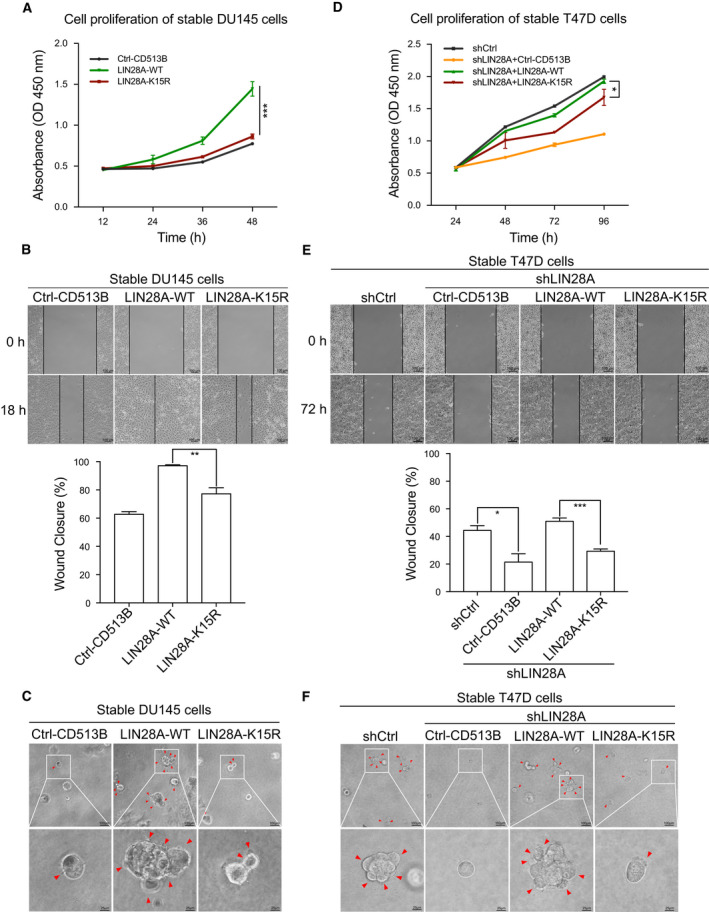
Mutation K15R of LIN28A abolishes its oncogenic roles. (A, D) Mutation K15R of LIN28A inhibits cancer cell proliferation. CCK8 cell proliferation assays were performed with DU145 (A) and T47D (D) stable cells. Error bars ± SEM represent three independent experiments with at least triplicate repeats. Differences between individual groups as indicated were analyzed using the two‐way ANOVA, and *P* values of < 0.05 (*) or < 0.01 (**) or < 0.001 (***) are considered significant. (B, E) SUMOylation of LIN28A promotes cancer cell migration. Wound‐healing assays were performed on DU145 (B) and T47D (E) stable cell lines. Images were captured at indicated time. The areas of the wound were calculated by imagej software, and the percentage of wound closure was presented by histogram. Error bars ± SD represent three independent experiments. Differences between individual groups as indicated were analyzed using the t‐test (two‐tailed and unpaired), and *P* values of < 0.05 (*), < 0.01 (**), or < 0.001 (***) are considered significant. (C, F) LIN28A‐K15R loses its ability in promoting cancer cell invasion. The 3D cell culture growth assay was performed to detect the invasive ability of DU145 (C) and T47D (F) stable cell lines. Images were captured at day 7 and shown in the large field of view (upper panel), and the representative images of 3D culture were shown (lower panel).

As known that LIN28A can promote proliferation, invasion, and metastasis of multiple cancer cells (Viswanathan and Daley, 2010), we wondered whether LIN28A SUMOylation plays a role in regulation of tumor progression. To this end, we generated stably expressing wild‐type (WT) or SUMO‐mutant K15R of LIN28A in DU145 cells, in which endogenous LIN28A is very low expressed (Fig. S5A). These DU145 stable cell lines were used to investigate the influences of LIN28A SUMOylation on cell proliferation and migration. As expected, ectopic expression of LIN28A‐WT significantly promoted cell proliferation compared with the control vector transfected. However, the expression of LIN28A‐K15R had little impact on cell proliferation (Fig. 3A). In order to assess whether SUMOylation of LIN28A influences the migration capacity of tumor cells, we performed woundhealing assay on DU145 stable cell lines. We found that the cell migration ability was enhanced by ectopic expression of LIN28A‐WT but not LIN28A‐K15R (Fig. 3B). Furthermore, we assessed the effect of LIN28A SUMOylation on the invasive ability of tumor cells by using the method of 3D cell culture, which mimic *in* *vivo* cell growth conditions. DU145‐LIN28A‐WT cells proliferated diffusely and exhibited dispersed morphology, reflecting the great ability to invade the extracellular matrix. In contrast, DU145‐LIN28A‐K15R and DU145‐Ctrl‐Vector cells grew into compact and round colonies (Figs 3C and S5B). These data suggest that LIN28A SUMOylation may be required to maintain the oncogenic function of LIN28A.

Furthermore, we took advantage of a breast cancer cell T47D with the high LIN28A expression to confirm above results. We generated T47D stable cell lines in which endogenous LIN28A was firstly knocked down by a short hairpin RNA targeting the 3′‐UTR of LIN28A (shLIN28A; Fig. S5C), and then, HA‐LIN28A‐WT or HA‐LIN28A‐K15R was re‐expressed by using the lentiviral expressing system (Fig. S5D). We performed above same experiments with these T47D stable cell lines. As expected, LIN28A knockdown inhibited the abilities of cell proliferation, migration, and invasion, which were restored by re‐expression of LIN28A‐WT but not LIN28A‐K15R in T47D‐shLIN28A cells (Figs 3D–F, and S5E). Thus, above results indicate that LIN28A SUMOylation plays an important role in regulating tumor cell progression. As known that LIN28A overexpression has been associated with low sensitivity to numerous cancer therapies including Cisplatin (Balzeau *et al*., 2017), so we performed the cell viability assay and showed that knockdown of LIN28A increased drug sensitivity to Cisplatin in T47D cells, which was rescued by re‐expression of LIN28A‐WT but not mutant LIN27A‐K15R (Fig. S6). This result suggests Lin28A SUMOylation is involved in drug resistance since Cisplatin down‐regulated SUMO1 modification of LIN28A (Fig. 1K‐L).

### 3.5 SUMOylation of LIN28A at K15 enhances its inhibition of let‐7 biogenesis

**Fig. 4:**
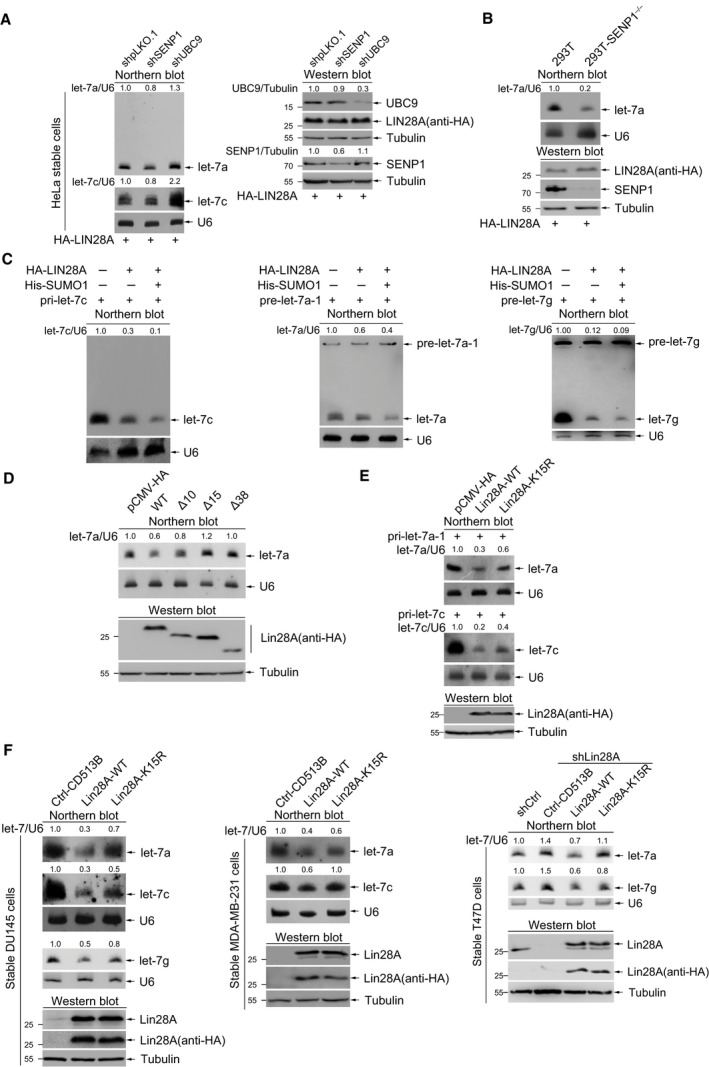
SUMOylation of LIN28A exacerbates its inhibition of let‐7 biogenesis. (A) Disrupting SUMOylation system interferences let‐7 biogenesis. HeLa‐shpLKO.1, HeLa‐shSENP1, and HeLa‐shUBC9 cells were transiently transfected with HA‐LIN28A. 48 h after transfection, the expression levels of endogenous let‐7a and let‐7c were detected by northern blotting with indicated probes. The knockdown efficiency of SENP1 and UBC9 was detected by western blot with indicated antibodies. The SENP1 and UBC9 bands were quantified by imagej software and normalized with Tubulin. (B) Stable knockout of SENP1 leads to down‐regulation of mature let‐7a levels. 293T cells and 293T *SENP1* ^−/−^ cells were transiently transfected with HA‐LIN28A. The expression level of endogenous let‐7a was analyzed by northern blotting. The effects of knockout of SENP1 were detected by western blotting with indicated antibodies. (C) SUMO1 modification of LIN28A enhances its inhibition of let‐7s biogenesis. 293T cells were co‐transfected with HA‐LIN28A and human pri‐let‐7c, pre‐let‐7a‐1, or pre‐let‐7g, with or without His‐SUMO1, as indicated. 48 h after transfection, RNAs were extracted and separated on 20% polyacrylamide 8 m urea gels. U6 RNA was used as a control. (D) Truncated forms lacking K15 do not inhibit let‐7a biogenesis. 293T cells were transiently transfected with HA‐LIN28A and truncated forms as indicated. Northern blot was used to measure the expression levels of endogenous let‐7a. (E, F) Mutation K15R of LIN28A blocks its inhibition of let‐7 biogenesis. (E) 293T cells were transiently transfected with HA‐LIN28A or HA‐LIN28A‐K15R, along with pri‐let‐7a‐1 or pri‐let‐7c. (F) Stable DU145, MDA‐MB‐231, and T47D‐shLIN28A expressing the control vector, HA‐LIN28A, or HA‐LIN28A‐K15R were used for northern blotting analyses of let‐7s. The expression levels of LIN28A and LIN28A‐K15R were detected by western blotting with anti‐HA and anti‐LIN28A antibodies. All of let‐7 bands were quantified by imagej software and normalized with U6.

LIN28A is a conserved RBP that directly down‐regulates let‐7 maturation, and aberrant regulation of the LIN28A‐let‐7 axis exists in human malignancies (Viswanathan *et al*., 2009; Wang *et al*., 2015). We hypothesized that SUMOylated LIN28A exerts oncogenic effects by regulating the LIN28A‐let‐7 axis. To confirm this hypothesis, we used shRNAs to knockdown SENP1 or UBC9 expression in HeLa cells, and then transfected HA‐LIN28A into these cells. Northern blotting results showed that endogenous let‐7a and let‐7c were somewhat decreased with the low efficiency of SENP1 knockdown in HeLa‐shSENP1 cells when compared with HeLa‐shCtrl cells (Fig. 4A). In contrast, knockdown of UBC9 increased the biogenesis of mature let‐7a and let‐7c (Fig. 4A). Simultaneously, we used the CRISPR‐Cas9 system to more efficiently knockout SENP1 in 293T cells and then ectopically expressed with HA‐LIN28A for determination the effect on let‐7 expression. As expected, depletion of SENP1 led to a decrease in let‐7a level (Fig. 4B). These results suggested that SUMOylation of LIN28A was involved in the regulation of let‐7 biogenesis. To further confirm this, pri‐let‐7c, pre‐let‐7a‐1, or pre‐let‐7g was co‐transfected with or without HA‐LIN28A and His‐SUMO1 into 293T cells to detect let‐7 biogenesis. Northern blotting results showed that SUMOylation enhanced the inhibition of let‐7a/7c/7g biogenesis mediated by LIN28A (Fig. 4C), revealing that SUMOylation of LIN28A promoted its repression on let‐7 maturation.

To more strongly support this concept, we next used the N‐terminal truncated forms and the SUMO‐site mutant K15R of LIN28A to test the effects on let‐7 biogenesis. We performed northern blotting analysis for the mature let‐7a levels in 293T cells ectopically expressed with LIN28A‐WT, LIN28A‐∆10, LIN28A‐∆15, or LIN28A‐∆38. As shown in Fig. 4D, compared to the control vector, two K15‐existing forms of LIN28A‐WT or LIN28A‐∆10 repressed, whereas the non‐K15 forms of LIN28A‐∆15 or LIN28A‐∆38 have no much effects on endogenous let‐7 biogenesis, suggesting that LIN28A truncated forms lacking the SUMO‐site K15 reduced the abilities in inhibiting let‐7a biogenesis. Furthermore, pri‐let‐7a or pri‐let‐7c was co‐transfected with LIN28A‐WT or LIN28A‐K15R following by northern blot analysis. The result showed that the efficiency in inhibition of let‐7a or let‐7c by LIN28A‐WT was higher than that by LIN28A‐K15R, which suggested the SUMO‐site mutation K15R of LIN28A reduced its inhibitory effects on let‐7a and let‐7c (Fig. 4E). Lastly, in addition to stable cell lines DU145 and T47D (used in Fig. 3), we also generated stable MDA‐MB‐231 cells (with low/no endogenous LIN28A expression) ectopically expressing LIN28A‐WT or LIN28A‐K15R. The results of northern blotting showed that the production of tested endogenous let‐7s (including let‐7a, let‐7c, and let‐7g) in ectopically re‐expressing LIN28A‐WT stable cells was more inhibited than those in re‐expressing LIN28A‐K15R stable cells (Fig. 4F). Collectively, our results demonstrate that SUMOylation of LIN28A at K15 promotes its inhibition of the production of mature let‐7s.

### 3.6 SUMOylation of LIN28A increases its binding to pre‐let‐7 in cells

**Fig. 5:**
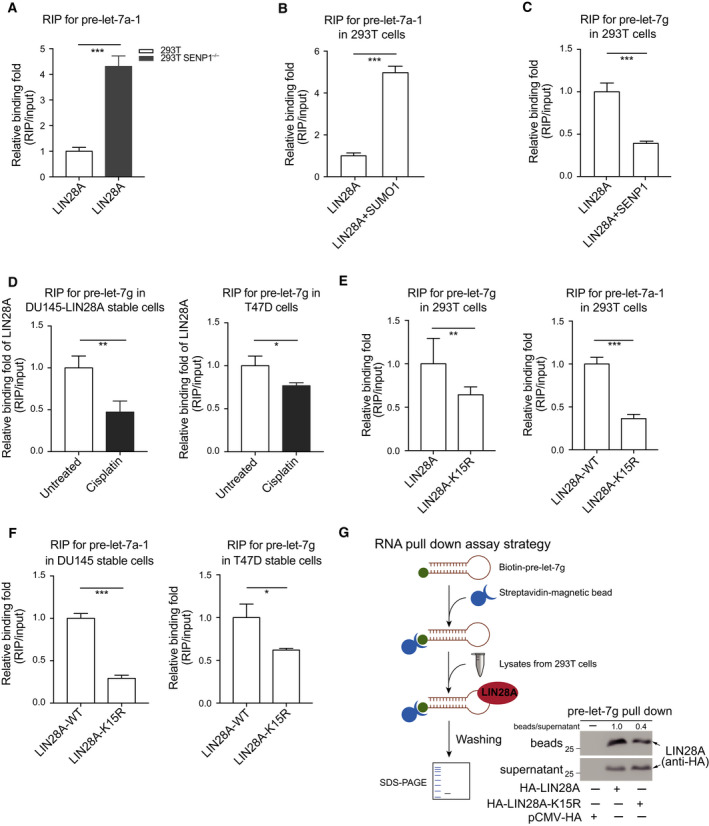
SUMOylation of LIN28A increases its binding to pre‐let‐7. (A) Knockout of SENP1 increases the interaction of LIN28A with pre‐let‐7a‐1. 293T cells and 293T *SENP1* ^−/−^ cells transfected with HA‐LIN28A and pre‐let‐7a‐1 were lysed for RIP assay. (B) SUMO1 modification enhances the association of LIN28A with pre‐let‐7a‐1. 293T cells transfected with indicated plasmids were used for RIP assays. (C) DeSUMOylation by SENP1 suppresses the interaction of LIN28A with pre‐let‐7g. 293T cells transfected with indicated plasmids were used for RIP assays. (D) Cisplatin suppresses the interaction of LIN28A with pre‐let‐7g. DU145 stably expressing HA‐LIN28A cells or T47D‐shLIN28A re‐expressing HA‐LIN28A stable cells were treated with Cisplatin (10 µm) for 12 h, and then, RIP assays were performed. (E, F) Pre‐let‐7s bound to LIN28A‐WT were much more than that to SUMO‐site mutant LIN28A‐K15R. 293T cells transfected with HA‐LIN28A or HA‐LIN28A‐K15R together with indicated pre‐let‐7 plasmids were used for RIP assays (E). RIP analysis of RNAs associated with HA‐LIN28A or HA‐LIN28A‐K15R from DU145 and T47D stable cell lines (F). RIP assays were carried out with anti‐HA (E) or anti‐LIN28A (F) antibody. (G) RNA pull‐down assay using biotinylated pre‐let‐7g and lysates from 293T cells transiently expressing HA‐LIN28A or HA‐LIN28A‐K15R. RNA‐bound fraction (beads) and unbound fraction (supernatant) were detected by western blotting with anti‐HA antibody. LIN28A bands were quantified by imagej software. The schematic diagram of RNA pull‐down assay is presented. RNAs extracted from IP complexes were analyzed by qRT–PCR, all RNA signals of RIP were normalized to those of input, then presented by relative binding fold. All data for qRT–PCR are presented as the mean ± SD with triplicates or quadruplicate sets, differences between individual groups as indicated were analyzed using the *t*‐test (two‐tailed and unpaired), and *P* values of < 0.05 (*), < 0.01 (**), <0.001 (***) are considered significant.

Next, we sought to explore the molecular mechanism underlying let‐7 processing inhibition mediated by SUMOylation of LIN28A. Since LIN28A inhibits biogenesis of let‐7 through directly binding to the TL region of pre‐let‐7 (Nam *et al*., 2011; Newman *et al*., 2008), we firstly compared the relative binding abilities of different SUMOylation degree of LIN28A with pre‐let‐7s. HA‐LIN28A was transfected into 293T cells or 293T SENP1^−/−^ cells, and then, a RIP assay was conducted by using antibodies against HA. The relative levels of pre‐let‐7a‐1 bound to LIN28A were quantified by qRT–PCR. The results showed that pre‐let‐7a‐1 bound to LIN28A at the higher SUMOylation status in 293T *SENP1* ^−/−^ cells was more than that of in 293T cells (Fig. 5A), suggesting that high SUMOylation enhanced the binding ability of LIN28A to pre‐let‐7a‐1. We also transfected LIN28A with or without SUMO1 or SENP1 into 293T and subsequently performed RIP assays, showing that the binding of pre‐let‐7a‐1 to LIN28A was enormously enhanced by increased SUMOylation (Fig. 5B); on the contrary, it was reduced by deSUMOylation (Fig. 5C). Moreover, we treated DU145 expressing LIN28A stable cells and T47D cells with Cisplatin (10 µm) for 12 h and performed RIP analyses for endogenous pre‐let‐7g. We observed a decline of pre‐let‐7g associated with LIN28A in both DU145 stable cells and T47D cells after the treatment of Cisplatin (Fig. 5D), which down‐regulated SUMOylation of LIN28A (as shown in Fig. 1J‐L). Collectively, these findings indicate that SUMOylation LIN28A can enhance its binding affinity with pre‐let‐7s.

In order to further assess the effects of SUMOylation at K15 on LIN28A binding to pre‐let‐7s, 293T cells were transfected with HA‐LIN28A or HA‐LIN28A‐K15R for RIP assays, showing that the relative level of endogenous pre‐let‐7g or pre‐let‐7a‐1 binding to LIN28A‐K15R was much less than that of ectopically expressed LIN28A‐WT (Fig. 5E). The similar results were obtained in DU145 expressing HA‐LIN28A‐WT or HA‐LIN28A‐K15R stable cell lines and T47D‐shLIN28A re‐expressing HA‐LIN28A‐WT or HA‐LIN28A‐K15R stable cell lines (Fig. 5F). Moreover, we generated biotinylated pre‐let‐7g by using a T7 transcribe system and conducted RNA pull**‐**down assay. Cell lysates from 293T transiently expressing HA‐LIN28A‐WT or HA‐LIN28A‐K15R were incubated with Streptavidin–Dynabeads‐coupled biotinylated pre‐let‐7g, and followed by washing steps and western blotting with anti‐HA antibody. The result showed that the recruitment of pre‐let‐7g to LIN28A‐WT was more than that to LIN28A‐K15R (Fig. 5G). Thus, above data demonstrate that SUMOylation of LIN28A increases its binding to pre‐let‐7 in cells.

### 3.7 SUMOylation of LIN28A augments its interaction with pre‐let‐7 *in vitro*

**Fig. 6:**
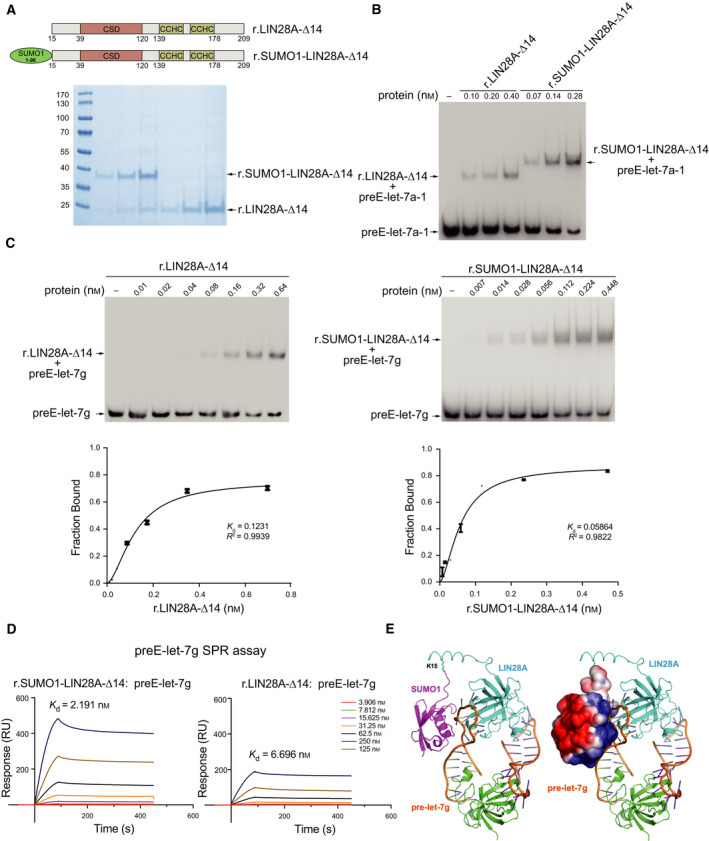
SUMOylation of LIN28A augments its affinity with pre‐let‐7 *in vitro*. (A) Top: Schematic maps of r.LIN28A‐∆14 and r.SUMO1‐LIN28A‐∆14 protein domains. Bottom: Coomassie Blue staining of purified r.LIN28A‐∆14 and r.SUMO1‐LIN28A‐∆14 proteins. (B, C) Binding of r.LIN28A‐∆14 and r.SUMO1‐LIN28A‐∆14 to synthetic preE‐let‐7a‐1 was assessed by EMSA with 5 nm of 5′‐end biotin‐labeled preE‐let‐7a‐1 (B) or preE‐let‐7g (C) and the indicated concentration of recombinant proteins. The band intensities were quantified by imagej software and presented as the fraction of bound preE‐let‐7g RNA in the plots. (D) SPR analysis of the direct binding of r.LIN28A‐∆14 and r.SUMO1‐LIN28A‐∆14 to synthetic preE‐let‐7g using an Biacore T200 instrument. The binding affinity was determined by global fitting to a Langmuir 1 : 1 binding model within the biacore evaluation software. (E) The crystal structures of SUMO1 (PDB: 4WJQ) and LIN28A‐preE‐Let‐7g (PDB: 3TS2) were blindly docked at ClusPro 2.0 docking server with a distance restrain of 20 Angstroms between Ca atoms of Gly96 of Sumo1 and Gln36 of LIN28A. The top solution from the server was selected for presentations here (left). The N‐terminal segment of LIN28A (residues 12–35), which was missing in the crystal structure, is modeled here as a helical structure with the sidechain of K15 (shown in sticks) covalently linked to the carboxyl group of Gly96 of SUMO1. The covalently linked SUMO1 (purple) on LIN28A (cyan) could readily interact with the backbone of preE‐let‐7g (brown) in the complex where the positively charged surface of SUMO1 (right) can form strong electrostatic interactions with the negatively charged phosphate groups of preE‐let‐7g. Therefore, the SUMOylated LIN28A would have higher binding affinity toward preE‐let‐7g. The electrostatic surface (right) of Sumo1 (with positively charged area shown in blue and negatively charged area shown in red) was calculated with software APBS, and the cartoon of the structures was generated using software pymol.

To further support the hypothesis that SUMOylated LIN28A is more favorable to bind to pre‐let‐7s, we purified LIN28A‐∆14 and SUMO1‐LIN28A‐∆14, a SUMO1 peptide (aa 1–96) fused on the N‐terminal of LIN28A‐∆14 to mimic the SUMOylated LIN28A *in vitro*, by using *E. coil* expression system (Fig. 6A). The relative binding affinities of the two recombinant proteins to chemically synthesized preE‐let‐7s were assessed by electrophoretic mobility shift assay (EMSA). After incubation of recombinant proteins with preE‐let‐7a‐1 labeled by biotin at its 5′‐terminus (biotin‐preE‐let‐7a‐1), complexes formed by biotin‐preE‐let‐7a‐1 and LIN28A‐∆14 or SUMO1‐LIN28A‐∆14 were differentiated by native PAGE. We detected that biotin‐preE‐let‐7a‐1 bound to SUMO1‐LIN28A‐∆14 was more than to LIN28A‐∆14 (Fig. 6B). Using the same EMSA conditions, we found that the dissociation constants (*K*_d_) of biotin‐preE‐let‐7g bound to LIN28A‐∆14 and SUMO1‐LIN28A‐∆14 fusion protein were ~ 0.12 and 0.06 nm, respectively, which demonstrated that SUMO1‐LIN28A‐∆14 fusion protein possessed the higher affinity to preE‐let‐7g (Fig. 6C). These data were further confirmed by a SPR assay, in which biotinylated preE‐let‐7g was captured on the surface of SA chip and series concentrations of LIN28A‐∆14 and SUMO1‐LIN28A‐∆14 were injected into the flow system, respectively. Association and dissociation were measured as response units upon binding of recombinant protein and its release in subsequent wash steps. The binding affinity was determined by global fitting to a Langmuir 1 : 1 binding model within the biacore evaluation software. We observed similar binding affinity patterns, with a *K*_d_ of 6.7 nm for the LIN28A‐∆14 and 2.2 nm for the SUMO1‐LIN28A‐∆14, respectively (Fig. 6D). To further dissect the interaction between SUMOylated‐LIN28A and pre‐let‐7, the crystal structures of SUMO1 (PDB: 4WJQ) and LIN28A‐pre‐let‐7g (PDB: 3TS2) were blindly docked at ClusPro 2.0 docking server with a distance restrain of 20 Angstroms between Ca atoms of Gly96 of SUMO1 and Gln36 of LIN28A. The top solution from the server was selected for presentations. The N‐terminal segment of LIN28A, which was missing in the crystal structure, is modeled here as a helical structure with the sidechain of K15 (shown in sticks) covalently linked to the carboxyl group of Gly96 of SUMO1. The covalently linked SUMO1 on LIN28A could readily interact with the backbone of pre‐let‐7g in the complex where the positively charged surface of SUMO1 could form strong electrostatic interactions with the negatively charged phosphate groups of pre‐let‐7g. Therefore, the SUMOylated LIN28A would have higher binding affinity toward pre‐let‐7g. The electrostatic surface of SUMO1 was calculated with software APBS, and the cartoon of the structures was generated using software pymol (Fig. 6E). Taken together, these data suggest that pre‐let‐7s preferentially associate with SUMOylated LIN28A.

### 3.8 LIN28A SUMOylation promotes pre‐let‐7 uridylation and inhibits pre‐let‐7 processing

**Fig. 7:**
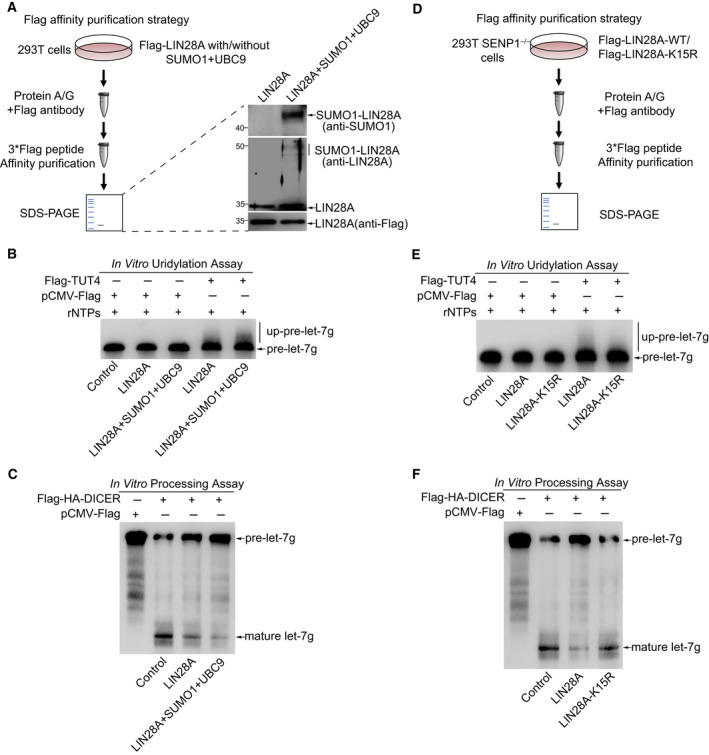
SUMOylation of LIN28A promotes pre‐let‐7 uridylation and inhibits pre‐let‐7 processing. (A, D) Schematic representation is shown for protein purification strategy from 293T cells transfected as indicated expressing plasmids. Purified Flag‐tagged LIN28A and SUMO1‐LIN28A (transfected with SUMO1/UBC9) proteins were immunoblotted with the indicated antibodies (A, right panel). (B, E) SUMOylation of LIN28A promotes pre‐let‐7 oligouridylation. For *in vitro* uridylation assay, purified Flag‐tagged LIN28A‐WT, SUMO1‐LIN28A (A), or LIN28A‐K15R (D) was incubated with *in vitro* transcribed uniformly biotin‐labeled pre‐let‐7g along with or without Flag‐TUT4, which was immunoprecipitated from 293T cells. After 30 min of reaction, RNAs were exacted from the reaction mixture and separated on 20% polyacrylamide 8 m urea gels, and then detected by northern blot. (C, F) SUMOylation of LIN28A inhibits pre‐let‐7 processing by DICER. For *in vitro* processing assay, transcribed *in vitro* uniformly biotin‐labeled pre‐let‐7g was incubated with purified Flag‐tagged LIN28A‐WT, SUMO1‐LIN28A (A), or LIN28A‐K15R (D) along with Flag‐HA‐DICER immunoprecipitated from 293T cells. After 60 min of reaction, RNAs were exacted from the reaction mixture and separated on 20% polyacrylamide 8 m urea gels and then detected by northern blot.

It has been reported that LIN28A can directly bind to the TL of pre‐let‐7 and simultaneously recruits the terminal TUTases TUT4/7, thus to induce oligouridylation of pre‐let‐7, which sequesters pre‐let‐7 from cleavage by DICER and subsequently promotes pre‐let‐7 degradation (Hagan *et al*., 2009; Heo *et al*., 2008; Heo *et al*., 2009). To explore the effect of the SUMOylated LIN28A on oligouridylation of pre‐let‐7 mediated by TUT4, we purified Flag‐tagged LIN28A and SUMO1‐LIN28A (by co‐transfection with SUMO1/UBC9) proteins by 3× Flag peptide system from 293T cells (Fig. 7A) and conducted *in vitro* uridylation assays and *in vitro* processing assays. Immunoprecipitated TUT4 from 293T cells and biotinylated pre‐let‐7g were incubated together with purified LIN28A or SUMO1‐LIN28A at 37 °C in reactions containing rNTPs for 30 min, and then, RNAs were purified from the reaction mixture and analyzed with 20% urea polyacrylamide gel. We observed the oligouridylation of pre‐let‐7g when purified LIN28A or SUMO1‐LIN28A was added to the reaction, and the uridylation was greatly enhanced by SUMO1‐LIN28A compared to that by LIN28A (Fig. 7B). It is also reported that the interaction between LIN28A and pre‐let‐7 can directly prevent the final processing of pre‐let‐7 mediated by DICER (Nam *et al*., 2011; Rybak *et al*., 2008), so we wondered whether SUMOylation of LIN28A is involved in DICER processing for let‐7 maturation. To address this, we then performed an *in vitro* pre‐let‐7 processing assay. Very similarly, we mixed Flag‐HA‐DICER immunoprecipitated from 293T cells and biotinylated pre‐let‐7g together with purified Flag‐tagged LIN28A or SUMO1‐LIN28A in reactions at 37 °C for 60 min, then RNAs were purified from the reaction mixture and analyzed with 20% urea polyacrylamide gel. Compared with LIN28A, SUMOylated LIN28A (SUMO1‐LIN28A) showed considerably more inhibition of DICER processing resulting in a sharp reduction of mature let‐7g (Fig. 7C).

To further test whether SUMOylation at K15 of LIN28A affects uridylation and processing of pre‐let‐7g, Flag‐LIN28A and Flag‐LIN28A‐K15R were purified from 293T *SENP1* ^−/−^ cells according to the similar strategy (Fig. 7D). As expected, the *in vitro* pre‐let‐7 uridylation assay showed the reduction in LIN28A SUMOylation by SUMO‐site mutation of K15R led to decrease in the uridylation of pre‐let‐7g compared to that of LIN28A‐WT (Fig. 7E). Moreover, the *in vitro* pre‐let‐7 processing assay showed that the mutation K15R of LIN28A diminished its inhibition of DICER processing, accompanied with more mature let‐7g production (Fig. 7F), which was consistent with our previous data (Fig. 4E,F). Collectively, these data demonstrated that the SUMOylation of LIN28A inhibited the maturation of let‐7 through increasing uridylation mediated by TUT4 and decreasing cleavage by DICER.

### 3.9 SUMOylation of LIN28A promotes tumorigenesis and tumor growth *in vivo* by enhancing inhibition of let‐7 biogenesis

**Fig. 8:**
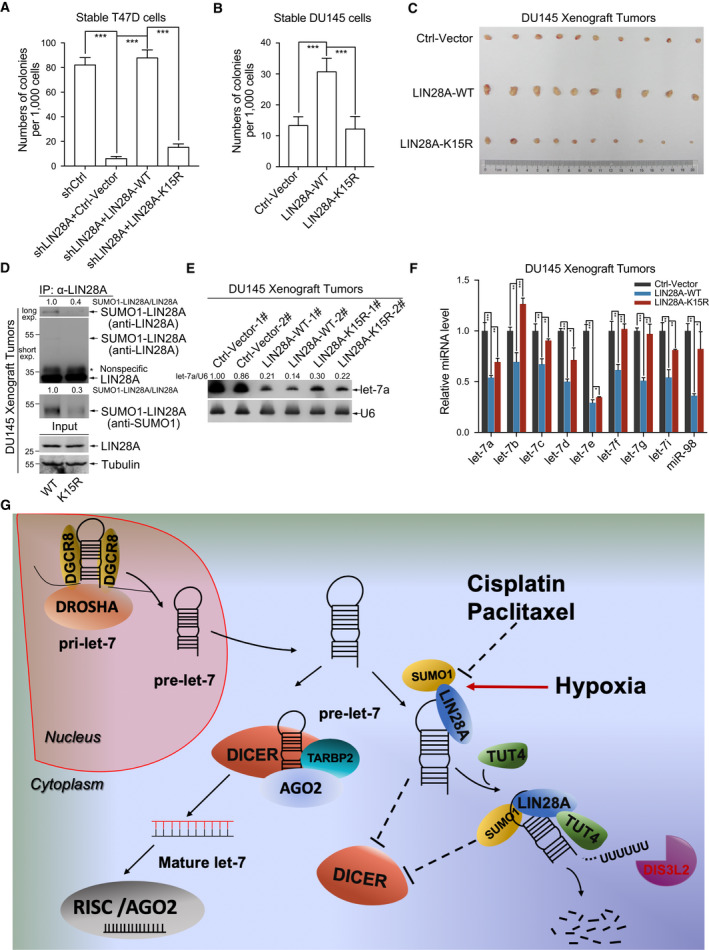
SUMOylation of LIN28A promotes tumorigenesis and tumor growth *in* *vivo*. (A, B) LIN28A‐K15R suppresses anchorage‐independent growth of cancer cells. T47D (A) or DU145 (B) stable cell lines as indicated were seeded in 2 mL of medium containing 10% FBS with 0.35% soft agarose at a density of 1000 cells per well and layered on 0.6% solidified agarose. After 21 days of culture, colonies were stained, and images were captured. The number of colonies was scored and graphically represented. Error bars ± SEM represent three independent experiments with at least triplicate repeats. (C) LIN28A‐K15R suppresses xenograft tumor growth *in* *vivo*. DU145 stably expressing the control vector, HA‐LIN28A‐WT, or HA‐LIN28A‐K15R cells (2.5 × 10^6^ cells/each) was subcutaneously injected into 5‐week‐old male BALB/c nude mice individually. Mice were killed after 4 weeks of injection. Tumors were dissected. (D) SUMOylation of LIN28A is stronger than that of LIN28A‐K15R in nude mice xenograft tumors. Xenograft tumors from LIN28A‐WT group and LIN28A‐K15R group were lysed in NEM‐RIPA buffer as described in the methods. The proteins were immunoprecipitated by anti‐LIN28A antibody, and then detected with anti‐SUMO1 and anti‐LIN28A antibodies. The SUMO1‐LIN28A and LIN28A (IP panels) band intensities were quantified by imagej software. The ratio of SUMO1‐LIN28A/LIN28A presents the intensity of SUMOylated LIN28A. (E) Northern blotting detection of endogenous let‐7a levels in xenograft tumors. Let‐7 band intensities were quantified by imagej software and normalized with U6. (F) qRT–PCR shows changes in the levels of endogenous mature let‐7s in xenograft tumors. U6 was used as controls for the mature miRNAs. All data are presented as the mean ± SD for qRT–PCR. Differences between individual groups as indicated were analyzed using the *t*‐test (two‐tailed and unpaired), and *P* values of < 0.05 (*), < 0.01 (**), or < 0.001 (***) are considered significant. (G) The proposed model of SUMOylated LIN28A inhibiting let‐7 maturation. The positively charged surface of SUMO1 can form strong electrostatic interaction with negatively charged phosphate groups of pre‐let‐7, which increases the binding affinity of LIN28A and pre‐let‐7. The intense interaction of SUMO1‐LIN28A‐pre‐let‐7 can efficiently recruit TUT4 to uridylate pre‐let‐7 and block the DICER processing of pre‐let‐7, thereby leading to degradation of pre‐let‐7 and consequently reducing mature let‐7 biogenesis. SUMOylation of LIN28A can be repressed by chemotherapy drugs including Cisplatin and PTX, but promoted by hypoxia.

We have presented that mutation K15R of LIN28A attenuated its roles in promoting cancer cell proliferation, migration, and 3D cell culture growth (Fig. 3) by retarding its inhibition of mature let‐7 biogenesis (Fig. 4), so we guessed that SUMOylation of LIN28A might also promote cellular transformation and tumorigenesis by enhancing inhibition of let‐7 biogenesis. To confirm this point, soft agar colony‐forming assays were performed with T47D‐shLIN28A re‐expressing HA‐LIN28A‐WT/‐K15R, DU145 stably expressing HA‐LIN28A‐WT/‐K15R cell lines and BPH1 (human prostate hyperplasia cells) stably expressing HA‐LIN28A‐WT/‐K15R cell lines (Fig. S7A). The results showed that mutation of K15R of LIN28A reduced the anchorage‐independent growth ability of DU145 cells (Figs 8A,B and S7B‐E). To further assess SUMOylation of LIN28A whether affects xenograft tumor growth *in* *vivo*, DU145 stable cell lines were injected subcutaneously into the flanks of nude mice. 30 days after injection, tumor xenografts were dissected and measured. The average sizes and weights of tumors in the HA‐LIN28A‐K15R group were significantly reduced compared to those of in the HA‐LIN28A‐WT group (Figs 8C and S7F), which was in line with the results of the colony‐formation assays. Furthermore, we detected SUMOylation of LIN28A in the xenograft tumors from LIN28A‐WT and LIN28A‐K15R groups, and showed that SUMO1 modification of LIN28A in LIN28A‐WT group was much stronger than that of in LIN28A‐K15R group (Fig. 8D). We also detected the let‐7a levels in xenografts by northern blotting, showing that the let‐7a levels in xenograft tumors of LIN28A‐K15R group were higher than that of in LIN28A‐WT group (Fig. 8E). More convincingly, we confirmed the same conclusion by using qRT–PCR detection of all the let‐7 family members in xenograft tumors (Fig. 8F). Taken together, these results confirm that SUMOylated LIN28A promotes tumor malignancy by repressing biogenesis of let‐7.

## 4 Discussion

LIN28A is an evolutionarily conserved RBP that plays an important role in regulating development, pluripotency, and tumorigenesis. In particular, LIN28A is re‐activated in overall frequency ~ 15% primary human tumors and human cancer cell lines, and promotes malignancy through down‐regulation of let‐7s (Viswanathan *et al*., 2009). However, studies on post‐translational regulations of LIN28A in response to cellular stresses and subsequent effects on let‐7 biogenesis are still incomprehensive. Although LIN28A has been characterized in 1997, the first PTM of LIN28A was reported until 2014 and found that PCAF directly interacts with and acetylates the CDS domain of LIN28A, which leads to a severe reduction in the LIN28A protein level. In consequence, the expression of let‐7a is increased, and this process can be specifically reversed by the deacetylase SIRT1 (Wang *et al*., 2014). In hESCs, SET7/9 mono‐methylates LIN28A at K135, the methylation of LIN28A increases its protein stability and significantly contributes to subcellular localization of LIN28A to the nucleus. Methylation of LIN28A stimulates multimerization of LIN28A with primary let‐7 (pri‐let‐7), thereby sequestering pri‐let‐7 in the nucleoli and blocking processing of let‐7 in a TUTase‐independent manner (Kim *et al*., 2014). More recently, it has been reported that a deubiquitinating enzyme ubiquitin‐specific protease 28 interacts with and stabilizes LIN28A protein to extend its half‐life, thus enhancing the LIN28A‐mediated inhibition of endogenous let‐7 processing (Haq *et al*., 2019). Here, we found that LIN28A was post‐translationally modified by SUMO1 at K15 *in* *vivo* and *in vitro*. In all the Ni^2+^‐NTA pull‐down assays for SUMOylation in 293T cells, we observed the SUMOylated LIN28A appeared as a doublet bands, but they were closely to each other, indicating that the upper band was not a di‐SUMOylated form of LIN28A since one SUMO molecular weight is ~ 15 kDa. One possibility is that LIN28A SUMOylation might enhance its other PTMs such as phosphorylation and acetylation by crosstalk, and thus, we would speculate that the lower band was modified by only SUMOylation, while the higher band might be modified by a combination of SUMOylation with phosphorylation or acetylation.

We found that SUMOylation of LIN28A contributed to its binding ability with pre‐let‐7, leading to a reduction in mature let‐7 production (Figs 4, 5, 6). SUMOylated LIN28A aggravated cancer cell proliferation, migration, invasion, malignancy transformation, and tumor growth *in* *vivo*, thereby modulating tumor progression (Figs 3,8, and S7). Most importantly, SUMOylation is strongly linked to cancer and one that seems to be upregulated in most of cancers (Seeler and Dejean, 2017). Cancer cells are subject to a variety of environmental stresses including genotoxic and oxidative stresses, which often rapidly influence many downstream target SUMOylation behaving as a crucial cellular antistress mechanism. We found that SUMOylation of LIN28A in cancer cells was greatly induced by hypoxia, whereas it was repressed by chemotherapy drugs (Figs 1I‐L and S3). Taken together, our data reveal a novel mechanism, by which SUMOylation of LIN28A may be essential for activation of the LIN28A‐let‐7 pathway in cancer cells in response to stresses that have guiding significance for clinical treatment of cancer.

We have revealed by a series of studies that SUMOylation is implicated in the regulation of miRNA pathways and tumorigenesis by multiple mechanisms. For examples, TARBP2 is SUMOylated at K52, which recruits AGO2 to constitute the RNA‐induced silencing complex‐loading complex (RLC) and promotes more pre‐miRNAs to load into the RLC, thus increasing the gene‐silencing efficiency (Chen *et al*., 2015). DGCR8 is a major component of microprocessor that can be SUMOylated at two distinct sites K259 and K707 exhibiting opposite functions in cancer cells. SUMOylation at K259 of DGCR8 is promoted by p14ARF and then causes DGCR8 nuclear retention, which is required for DGCR8 function in the processing of pri‐miRNAs into pre‐miRNAs and suppressing tumorigenesis (Zhu *et al*., 2016). However, SUMOylation at K707 of DGCR8 controls direct function of some pri‐miRNAs on gene silencing and promotes tumorigenesis (Zhu *et al*., 2015). Moreover, KHSRP, a single‐stranded RBP existed in the DROSHA/DGCR8 complex, is SUMOylated at K87. SUMOylation of KHSRP inhibits its interaction with the pri‐miRNA and DROSHA/DGCR8 complex and promotes its subcellular location in cytoplasm. SUMOylated KHSRP also blocks processing of a subset of pri‐miRNAs harboring short G‐rich stretches in their TL, which results in the down‐regulation of TL‐G‐Rich miRNAs such as let‐7 family and consequential tumorigenesis (Yuan *et al*., 2017). In this study, we found that the covalently linked SUMO1 on K15 of LIN28A readily interacted with the backbone of pre‐let‐7g in the complex, where the positively charged surface of SUMO1 was able to form strong electrostatic interactions with the negatively charged phosphate groups of pre‐let‐7g. Therefore, the LIN28A and linked SUMO1 protein formed a clip that stably held the pre‐let‐7g in the middle (Fig. 6E), contributing to a higher binding affinity with pre‐let‐7g. The consequences of intensive interaction between SUMOylated LIN28A and pre‐let‐7 were clearly reflected in the much more efficient 3′‐oligouridylation together with the weakened DICER processing of pre‐let‐7 (Fig. 7). The suppressions of LIN28A on DICER processing are involved in two aspects; on the one hand, the CCHC zinc fingers of LIN28A dimerize on the GGAG motif of pre‐let‐7 where adjacent to the DICER cleavage site; as a result, DICER might be unable to recognize its substrates properly and fail to process pre‐let‐7 (Nam *et al*., 2011). On the other hand, oligouridylation by TUT4/TUT7 elongates the 3′‐terminal of pre‐let‐7 resulting in resistance to DICER cleavage. Further studies are required to distinguish the potential mechanism responsible for the specific involvement of DICER. The stronger interaction between SUMOylated LIN28A and pre‐let‐7, together with subsequent events amplified the efficiency of LIN28A‐mediated suppression of let‐7 biogenesis. The main SUMO‐site mutant LIN28A‐K15R led to accumulation of let‐7s in several cell lines; on the contrary, we observed sharp reduction in mature let‐7s in high LIN28A SUMOylation level (Fig. 4).

SUMOylation is a reversible and dynamic enzymatic cascade. Dynamic cycles of SUMOylation and de‐SUMOylation occur instantaneously within a few seconds. Up to date, more than 1000 SUMOylated proteins both in nucleus and in cytoplasm have been identified, and the numbers keep increasing (Geiss‐Friedlander and Melchior, 2007; Hendriks and Vertegaal, 2016). SUMOylation has large effects although most SUMO substrates appear to be SUMOylated to a small percentage at steady state (Geiss‐Friedlander and Melchior, 2007). The dysregulated SUMOylation has been involving in multiple biological events including tumorigenesis (Gong *et al*., 2017). Abnormal LIN28A activation has been identified in multiple cancers and associated with advanced cancer grade and poor prognosis. LIN28A is implicated in oncogenic functions such as promoting cell proliferation, migration, and invasion *via* inhibition of let‐7 biogenesis with subsequent activation of let‐7 targets such as *HMGA2*, *RAS*, *c‐MYC,* and cell‐cycle‐related factors (Chen *et al*., 2014; Sampson *et al*., 2007; Thornton and Gregory, 2012). Mutation of LIN28A SUMO‐site K15 to arginine decreased cell abilities of proliferation, migration, and anchorage‐independent growth, as well as xenograft tumor growth in nude mice (Figs 3,8 and S5B,E and S7). The expression levels of let‐7s were increased in these LIN28A‐K15R xenografts, indicating SUMOylation of LIN28A promotes tumor progress by suppressing let‐7 biogenesis (Fig. 8E,F). This finding was consistent with that SUMOylation of LIN28A can be induced upon hypoxia (Fig. 1I), which is often linked to poor patient outcomes (Hsieh *et al*., 2013; Walsh *et al*., 2014). LIN28A expression and let‐7 deprivation have been reported to be associated with resistance to numerous cancer therapies (Balzeau *et al*., 2017). Our previous study indicated that SUMOylation of METTL3 can be altered by chemotherapy drugs Camptothecin, Cisplatin, Doxorubicin, and Etoposide (Du *et al*., 2018). Intriguingly, SUMOlyation of LIN28A was reduced by Cisplatin and PTX (Figs 1J‐L and S3). Therefore, SUMOylation of LIN28A might be as a new strategy for tumor‐targeted therapy.

## 5 Conclusions

In summary, our findings shed light on a novel mechanism that SUMOylation of Lin28A is required for activation of the Lin28A‐let‐7 pathway in response to cancer cellular environment stresses, which could have implications for cancer prognosis and therapy.

## Conflict of interest

The authors declare no conflict of interest.

## Author contributions

JD, HZ, and RC performed most of the experiments; HY, XZ, YW, and JH helped with all experiments; AZ and ZS performed and analyzed Fig. 6A,D,E; JY and JD wrote the manuscript. All authors read and approved the final manuscript.

## Supporting information


**Fig. S1** **.** LIN28A is mainly SUMOylated by SUMO1.
**Fig. S2** **.** LIN28A is expressed in a subset of cancer cell lines and human tumor tissue.
**Fig. S3** **.** Paclitaxel and cisplatin down‐regulates SUMOylation of LIN28A.
**Fig. S4** **.** Other lysines at LIN28A are not SUMO sites.
**Fig. S5** **.** Western blot analysis of stable cell lines and statistical analysis of 3D culture growth.
**Fig. S6** **.** SUMO site mutant K15R increases the drug sensitivity of T47D cells to cisplatin.
**Fig. S7** **.** LIN28A‐K15R suppresses anchorage‐independent growth of cells and xenograft tumor growth.
**Table S1** **.** Primers for construction of plasmids and shRNAs.
**Table S2** **.** Sequences for RNA labeling and preE‐let‐7s.
**Table S3** **.** Sequences of probes for Northern Blot.
**Table S4** **.** Primers of qRT‐PCR.

## Data Availability

All data required to evaluate the conclusions of the paper are present in the main text or the Supplementary Materials of the paper.
