# Supplementary material for: SUMOylation modulates the LIN28A‐let‐7 signaling pathway in response to cellular stresses in cancer cells
Source: Mol Oncol. 2020 Jun 1;14(9):2288–312. doi: 10.1002/1878-0261.12694 (PMC7463354; doi:10.1002/1878-0261.12694)
Supplement: Supplementary file 1 — Fig. S1 . LIN28A is mainly SUMOylated by SUMO1. Fig. S2 . LIN28A is expressed in a subset of cancer cell lines and human tumor tissue. Fig. S3 . Paclitaxel and cisplatin down‐regulates SUMOylation of LIN28A. Fig. S4 . Other lysines at LIN28A are not SUMO sites. Fig. S5 . Western blot analysis of stable cell lines and statistical analysis of 3D culture growth. Fig. S6 . SUMO site mutant K15R increases the drug sensitivity of T47D cells to cisplatin. Fig. S7 . LIN28A‐K15R suppresses anchorage‐independent growth of cells and xenograft tumor growth. Table S1 . Primers for construction of plasmids and shRNAs. Table S2 . Sequences for RNA labeling and preE‐let‐7s. Table S3 . Sequences of probes for Northern Blot. Table S4 . Primers of qRT‐PCR. [file MOL2-14-2288-s001.pdf]

SUMOylation modulates the LIN28A-let-7 signaling pathway in response to cellular stresses in cancer cells

Jinzhuo Dou[1#], Hailong Zhang[1#], Ran Chen[1#], Zimei Shu[3], Haihua Yuan[5], Xian Zhao[1], Yanli Wang[1], Jian Huang[1], Aiwu Zhou [4]*, Jianxiu Yu[1, 2, 3]*

Supplemental Information includes:

7 figures and 4 tables.

Supplementary Figure Legend

Fig. S1. LIN28A is mainly SUMOylated by SUMO1. Lysates from 293T transfected with HA-LIN28A and His-SUMO1, -SUMO2 or -SUMO3 were pulldown with Ni^{2+}-NTA resin for SUMOylation assay following with Western blotting by indicated antibodies. The SUMO-LIN28A bands were quantified by ImageJ software.

Fig. S2. LIN28A is expressed in a subset of cancer cell lines and human tumor tissue.. (A) Western blot analysis of LIN28A protein in extracts of human cancer cell lines. (B) QRT-PCR analysis of LIN28A mRNA expression in human cancer cell lines. (C) LIN28A expression in human LUAD tumor tissue and adjacent normal tissue. N stands for adjacent normal tissue and T stands for tumor tissue.

Fig. S3. Paclitaxel and Cisplatin down-regulates SUMOylation of LIN28A. (A) Paclitaxel (PTX) down-regulates SUMOylation of LIN28A. 293T cells transfected HA-LIN28A and His-SUMO1 were treated with Paclitaxel (30 μM) for indicated times. Cells were lysed for precipitation with Ni^{2+}-NTA resin. Western blot was performed with the indicated antibodies. The SUMO-LIN28A bands were quantified by ImageJ software. (B-C) Cisplatin and Paclitaxel reduce SUMOylation of endogenous LIN28A. After 6 h treatment with Cisplatin (10 μM) (B) or 12 h treatment with Paclitaxel (30 μM) (C), T47D cells were lysed for IP with anti-LIN28A antibody, followed by Western blot with anti-SUMO1 and anti-LIN28A

antibodies. The SUMO1-LIN28A and LIN28A (IP panels) bands were quantified by ImageJ software. The ratio of SUMO1-LIN28A/LIN28A presents the intensity of SUMOylated LIN28A.

Fig. S4. Other lysines at LIN28A are not SUMO sites. HA-LIN28A-WT or different HA-LIN28A-mutants and His-SUMO1 were co-transfected into 293T cells. Cells were lysed for Ni^{2+}-NTA pull down, followed by western blotting with indicated antibodies.

Fig. S5. Western blotting analysis of stable cell lines and statistical analysis of 3D culture growth. (A) Western blot analysis of LIN28A in extracts of DU145 stably expressing HA-LIN28A or HA-LIN28A-K15R cell lines with indicated antibodies. (B, E) Statistical analysis of 3D culture growth of DU145 (B) and T47D (E) stable cell lines. The number of colonies with diffuse tumor sphere was counted and the ratio of the former to the number of total colonies was presented by histogram. Error bars ±SEM represent three independent experiments. Differences between individual groups as indicated were analyzed using the t-test (two-tailed and unpaired), and P values of <0.05 (*), < 0.01 (**) or < 0.001 (***) are considered significant. (C) Western blot analysis of LIN28A in extracts of stable cell lines T47D with shLIN28A-CDS and shLIN28A-3'UTR with indicated antibodies. (D) Western blot analysis of LIN28A in extracts of stable cell lines T47D-shLIN28A re-expressing HA-LIN28A or HA-LIN28A-K15R with indicated antibodies.

Fig. S6. SUMO site mutant K15R increases the drug sensitivity of T47D cells to Cisplatin. T47D stable cells were treated with Cisplatin for 48 h and cell viability was determined using the CCK8 assay. Error bars ±SEM represent three independent experiments. Differences between individual groups as indicated were analyzed using the t-test (two-tailed and unpaired), and P values of <0.05 (*), < 0.01 (**) or < 0.001 (***) is considered significant.

Fig. S7. LIN28A-K15R suppresses anchorage-independent growth of cells and xenograft tumor growth. (A) Western blot analysis of LIN28A in extracts of BPH1 stable cell lines with indicated antibodies. (B-C) Stable T47D-shLIN28A re-expressing HA-LIN28A or HA-LIN28A-K15R cell lines (B) or DU145 stably expressing HA-LIN28A or HA-LIN28A-K15R cell lines (C) were seeded in 2 ml of medium containing 10% FBS with 0.35% soft agarose at a density of 1,000 cells per well and layered on 0.6% solidified agarose. After 21 days of culture, colonies were stained, and images were captured. (D-E) BPH1 stably expressing HA-LIN28A or HA-LIN28A-K15R cell lines were seeded in 2 ml of medium containing 10% FBS with 0.35% soft agarose at a density of 5,000 cells per well and layered on 0.6% solidified agarose. After 21 days of culture, colonies were stained, and images were captured (D), the number of colonies was scored and graphically represented (E). Error bars ±SEM represent three independent experiments with at least triplicate repeats. (F) DU145 xenograft tumors weight related to Fig. 8C.

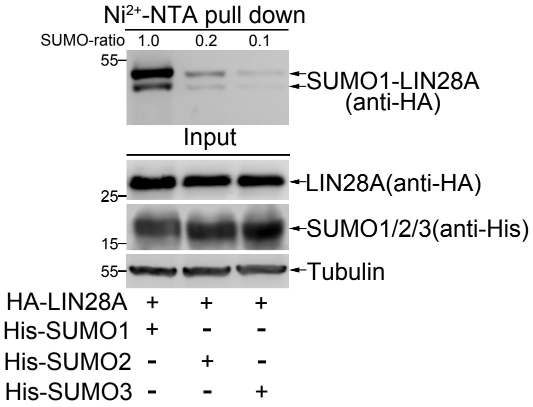

Ni2+-NTA pull down
SUMO-ratio 1.0 0.2 0.1
55
SUMO1-LIN28A
(anti-HA)
Input
LIN28A(anti-HA)
25
SUMO1/2/3(anti-His)
15
55
Tubulin
HA-LIN28A + + +
His-SUMO1 + - -
His-SUMO2 - + -
His-SUMO3 - - +

Dou_Fig. S2

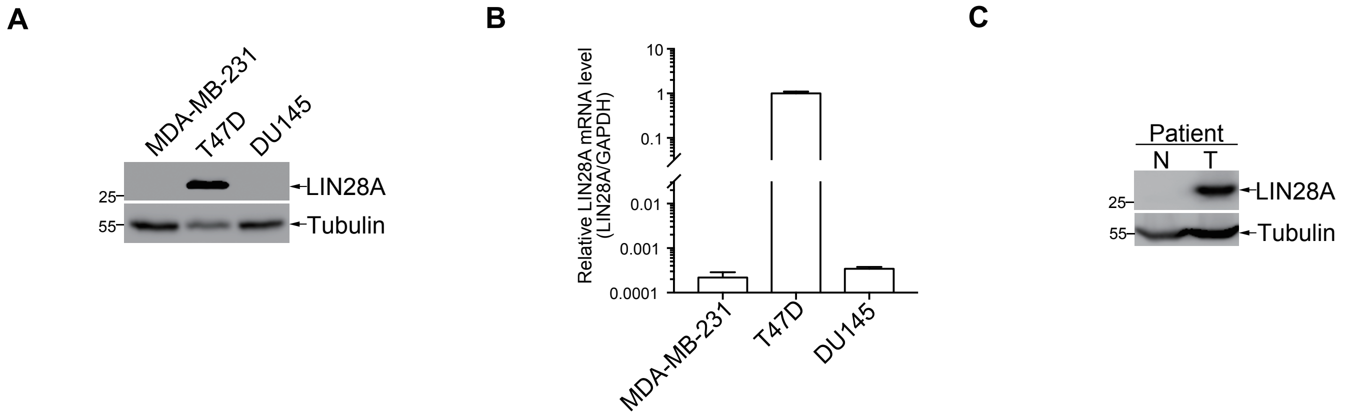
A
MDA-MB-231
T47D
DU145
25
55
LIN28A
Tubulin
B
Relative LIN28A mRNA level
(LIN28A/GAPDH)
10
1
0.1
0.01
0.001
0.0001
MDA-MB-231
T47D
DU145
C
Patient
N
T
25
55
LIN28A
Tubulin

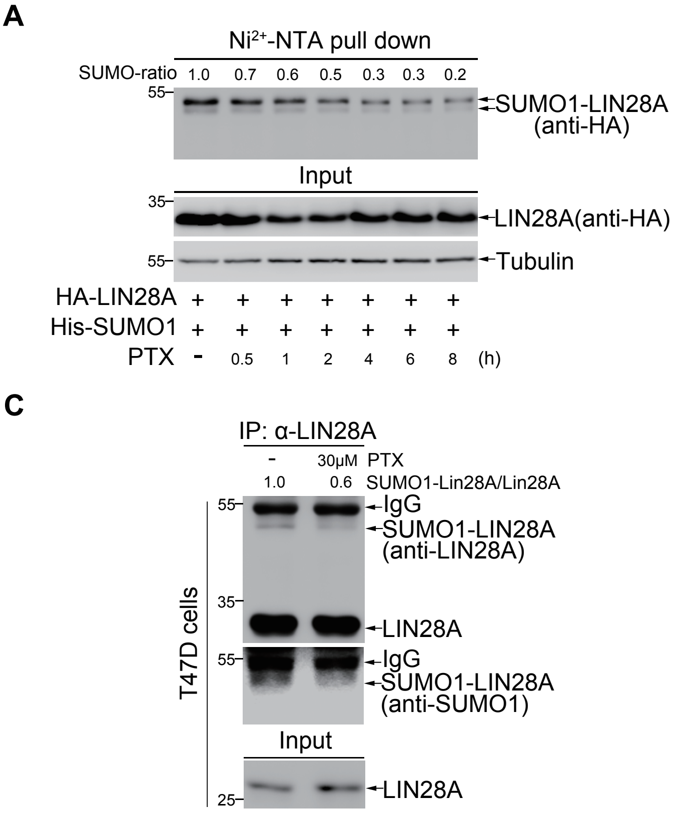
A
Ni2+-NTA pull down
SUMO-ratio 1.0 0.7 0.6 0.5 0.3 0.3 0.2
55
SUMO1-LIN28A (anti-HA)
Input
35
LIN28A(anti-HA)
55
Tubulin
HA-LIN28A + + + + + + +
His-SUMO1 + + + + + + +
PTX - 0.5 1 2 4 6 8 (h)
B
IP: α-LIN28A
- + Cisplatin
1.0 0.6 SUMO1-LIN28A/LIN28A
long exp.
55
short exp.
35
SUMO1-LIN28A (anti-LIN28A)
* Nonspecific
LIN28A
1.0 0.8 SUMO1-LIN28A/LIN28A
55
SUMO1-LIN28A (anti-SUMO1)
Input
LIN28A
Tubulin
T47D cells
C
IP: α-LIN28A
- 30μM PTX
1.0 0.6 SUMO1-Lin28A/Lin28A
55
IgG
SUMO1-LIN28A (anti-LIN28A)
35
LIN28A
55
IgG
SUMO1-LIN28A (anti-SUMO1)
Input
25
LIN28A
T47D cells

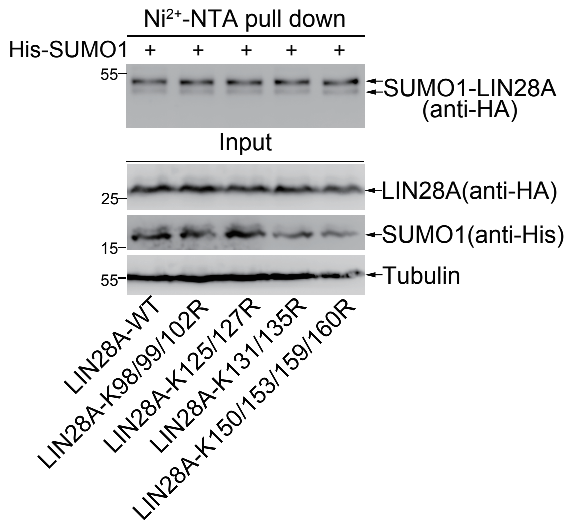
Ni2+-NTA pull down
His-SUMO1
+ + + + +
55
SUMO1-LIN28A (anti-HA)
Input
25
LIN28A(anti-HA)
15
SUMO1(anti-His)
55
Tubulin
LIN28A-WT
LIN28A-K98/99/102R
LIN28A-K125/127R
LIN28A-K131/135R
LIN28A-K150/153/159/160R

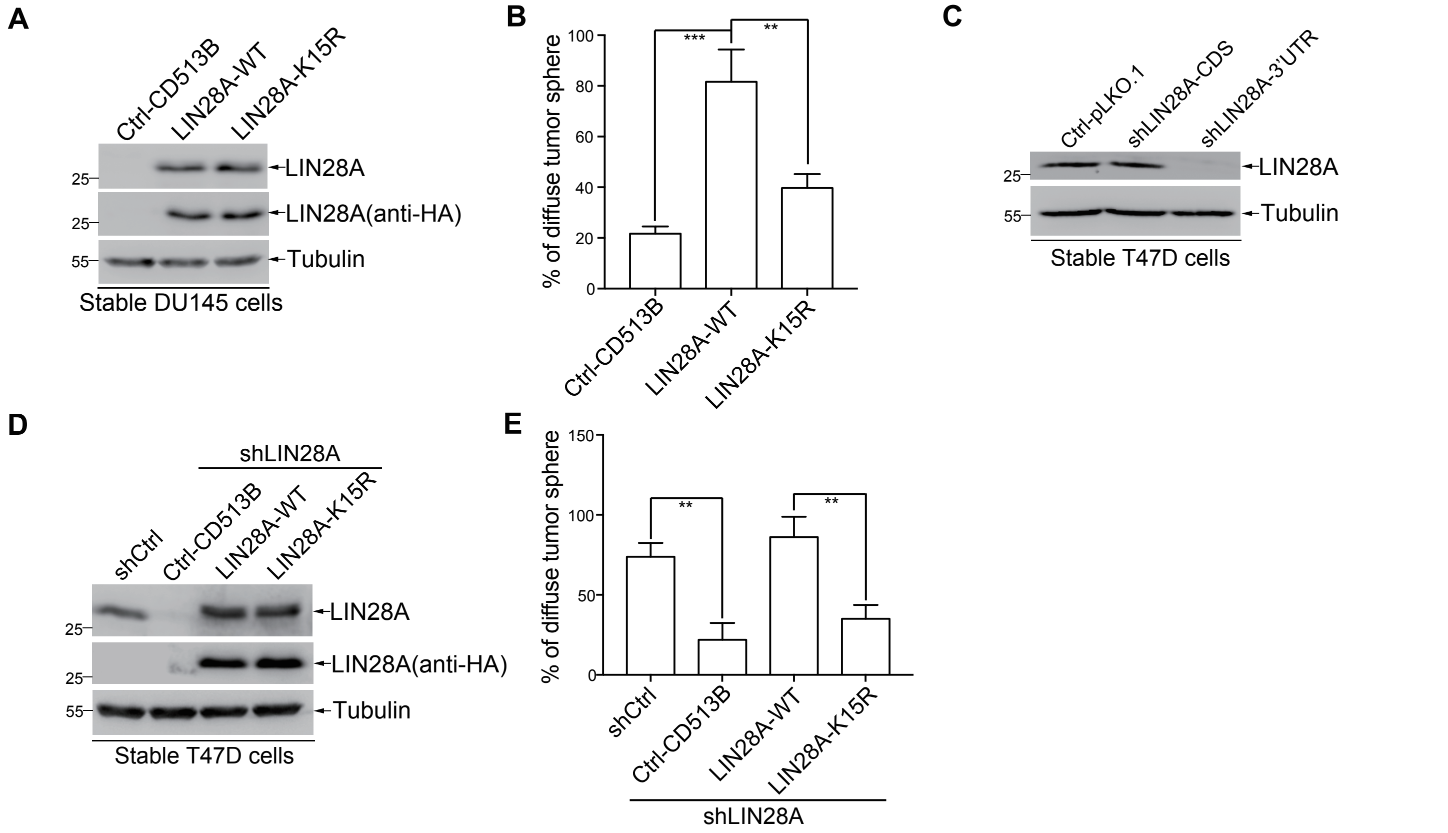
A
Ctrl-CD513B
LIN28A-WT
LIN28A-K15R
25
LIN28A
25
LIN28A(anti-HA)
55
Tubulin
Stable DU145 cells
B
% of diffuse tumor sphere
100
80
60
40
20
0
***
**
Ctrl-CD513B
LIN28A-WT
LIN28A-K15R
C
Ctrl-pLKO.1
shLIN28A-CDS
shLIN28A-3'UTR
25
LIN28A
55
Tubulin
Stable T47D cells
D
shLIN28A
shCtrl
Ctrl-CD513B
LIN28A-WT
LIN28A-K15R
25
LIN28A
25
LIN28A(anti-HA)
55
Tubulin
Stable T47D cells
E
% of diffuse tumor sphere
150
100
50
0
**
**
shCtrl
Ctrl-CD513B
LIN28A-WT
LIN28A-K15R
shLIN28A

Dou_Fig. S6

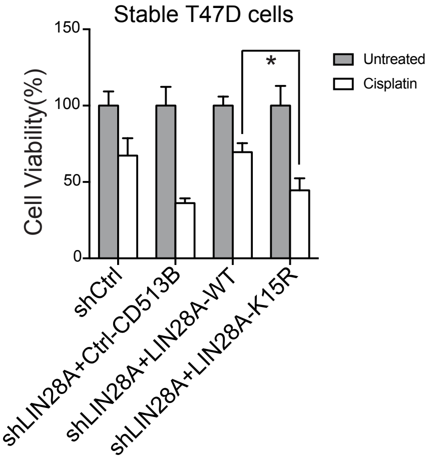
Stable T47D cells
Cell Viability(%)
150
100
50
0
*
Untreated
Cisplatin
shCtrl
shLIN28A+Ctrl-CD513B
shLIN28A+LIN28A-WT
shLIN28A+LIN28A-K15R

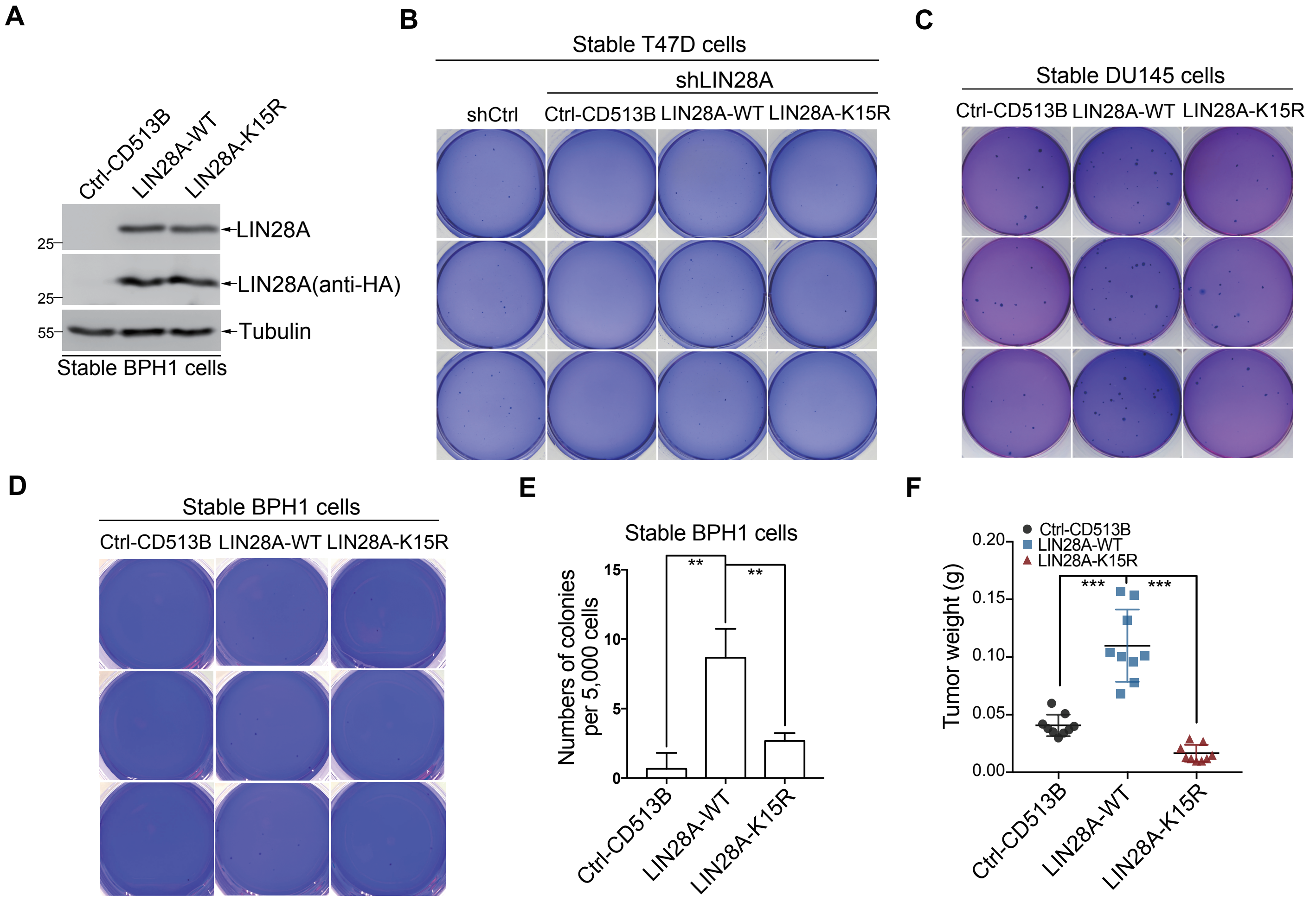
A
Ctrl-CD513B
LIN28A-WT
LIN28A-K15R
25
LIN28A
25
LIN28A(anti-HA)
55
Tubulin
Stable BPH1 cells
B
Stable T47D cells
shLIN28A
shCtrl
Ctrl-CD513B LIN28A-WT LIN28A-K15R
C
Stable DU145 cells
Ctrl-CD513B LIN28A-WT LIN28A-K15R
D
Stable BPH1 cells
Ctrl-CD513B LIN28A-WT LIN28A-K15R
E
Stable BPH1 cells
Numbers of colonies per 5,000 cells
15
10
5
0
**
**
Ctrl-CD513B
LIN28A-WT
LIN28A-K15R
F
Ctrl-CD513B
LIN28A-WT
LIN28A-K15R
Tumor weight (g)
0.20
0.15
0.10
0.05
0.00
***
***
Ctrl-CD513B
LIN28A-WT
LIN28A-K15R

Table S1. Primers for construction of plasmids and shRNAs

Primer	Sequence (5´-3´)
pCMV-HA-Lin28A-FWD	CGGAATTCGGATGGGCTCCGTGTCCAACCA
pCMV-HA-Lin28A-REV	CGGGGTACCTCAATTCTGTGCCTCCGGGA
pCMV-Myc-Lin28A-FWD	CGGAATTCGGATGGGCTCCGTGTCCAACCA
pCMV-Myc-Lin28A-REV	CGGGGTACCTCAATTCTGTGCCTCCGGGA
CD513B-HA-Lin28A-FWD	CGCGGATCCGCCACCATGTACCCATACGATGTTCC AGATTACGCTATGGGCTCCGTGTCCAACCAGC
CD513B-HA-Lin28A-REV	ATAAGAATGCGGCCGCTCAATTCTGTGCCTCCGGGA
pGEX-4T-1-Lin28A-FWD	CGCGGATCCATGGGCTCCGTGTCCAACCA
pGEX-4T-1-Lin28A-REV	ATTTGCGGCCGCTCAATTCTGTGCCTCCGGGA
Flag-Lin28A-FWD	CCGGAATTCATGGGCTCCGTGTCCAACCA
Flag-Lin28A-REV	CCCAAGCTTTCAATTCTGTGCCTCCGGGA
shLin28A-CDS-FWD	CCGGTAGAGCATGCAGAAGCGCAGATCTCGAGAT CTGCGCTTCTGCATGCTCTTTTTTG
shLin28A-CDS-REV	AATTCAAAAAAGAGCATGCAGAAGCGCAGATCTC GAGATCTGCGCTTCTGCATGCTCTA
shLin28A-3’UTR-FWD	CCGGTTAGTTGGCACTGCCATGTATCCTCGAGGAT ACATGGCAGTGCCAACTATTTTTG
shLin28A-3’UTR -REV	AATTCAAAAATAGTTGGCACTGCCATGTATCCTCG AGGATACATGGCAGTGCCAACTAA
pGreenpuropre-let-7a-1-FWD	GATCCGTGAGGTAGTAGGTTGTATAGTTTTAGGGT CACACCCACCACTGGGAGATAACTATACAATCTAC TGTCTTTCCTTTTTG
pGreenpuro pre-let-7a-1-REV	AATTCAAAAAGGAAAGACAGTAGATTGTATAGTTA TCTCCCAGTGGTGGGTGTGACCCTAAAACTATACA ACCTACTACCTCACG
pGreenpuro pre-let-7c-FWD	GATCCGTGAGGTAGTAGGTTGTATGGTTTAGAGTT ACACCCTGGGAGTTAACTGTACAACCTTCTAGCTT TCCCTTTTTG

pGreenpuro pre-let-7c-REV	AATTCAAAAAGGGAAAGCTAGAAGGTTGTACAGT TAACTCCCAGGGTGTAACTCTAAACCATACAACCT ACTACCTCACG
pGreenpuro pre-let-7g-FWD	GATCCGTGAGGTAGTAGTTTGTACAGTTTGAGGGT CTATGATACCACCCGGTACAGGAGATAACTGTACA GGCCACTGCCTTGCCTTTTTG
pGreenpuro pre-let-7g-REV	AATTCAAAAAGGCAAGGCAGTGGCCTGTACAGTT ATCTCCTGTACCGGGTGGTATCATAGACCCTCAAA CTGTACAAACTACTACCTCACG

Table S2. Sequences for RNA labeling and preE-let-7s

Primer	Sequence (5´-3´)
pUC57-pre-let-7g	TTAATACGACTCACTATAGGGAGACTACTACCTCACTGATGA GTCCGTGAGGACGAAACGGTACCCGGTACCGTCTGAGGTAG TAGTTTGTACAGTTTGAGGGTCTATGATACCACCCGGTACAG GAGATAACTGTACAGGCCACTGCCTTGC
cis-pre-let-7g-FWD	TTAATACGACTCACTATAGGGAGA
cis-pre-let-7g-REV	GCAAGGCAGTGGCCTGTACAG
preE-let-7a-1	UUAGGGUCACACCCACCACUGGGAGAUAA
preE-let-7g	UGAGGGUCUAUGAUACCACCCGGUACAGGAGAUAA

Table S3. Sequences of probes for Northern Blot

Probe	Sequence (5´-3´)
U6	TGTGCTGCCGAAGCGAGCAC
let-7a	AACTATACAACCTACTACCTCA
let-7c	AACCATACAACCTACTACCTCA
let-7g	ACTGTACAAACTACTACCTCA

Table S4. Primers of qRT-PCR

Primer	Sequence(5´-3´)

let-7a/7d/7e/7f-RT	GTCGTATCCAGTGCAGGGTCCGAGGTATTCGCA CTGGATACGACAACTAT
let-7b-RT	GTCGTATCCAGTGCAGGGTCCGAGGTATTCGCA CTGGATACGACAACCAC
let-7c-RT	GTCGTATCCAGTGCAGGGTCCGAGGTATTCGCA CTGGATACGACAACCAT
let-7g-RT	GTCGTATCCAGTGCAGGGTCCGAGGTATTCGCA CTGGATACGACAACTGT
let-7i-RT	GTCGTATCCAGTGCAGGGTCCGAGGTATTCGCA CTGGATACGACAACAGC
miR98-RT	GTCGTATCCAGTGCAGGGTCCGAGGTATTCGCA CTGGATACGACAACAAT
U6-RT	GTCGTATCCAGTGCAGGGTCCGAGGTATTCGCA CTGGATACGACTCAGTT
mature-miRNA-common-QRT-REV	GTGCAGGGTCCGAGGT
let-7a/7b/7c-QRT-FWD	GCCTGTGAGGTAGTAGGTTG
let-7d-QRT-FWD	GCCTGAGAGGTAGTAGGTTG
let-7e-QRT-FWD	GCCTGTGAGGTAGGAGGTTG
let-7f-QRT-FWD	GCCTGTGAGGTAGTAGATTG
let-7g-7i-QRT-FWD	GCCTGTGAGGTAGTAGTTTG
miR98-QRT-FWD	GCCTGTGAGGTAGTAAGTTG
pre-let7a-1-QRT-FWD	GGTAGTAGGTTGTATAGTTTTAGG
pre-let7a-1-QRT-REV	GAAAGACAGTAGATTGTATAGTT
pre-let7g-QRT-FWD	GGTAGTAGTTTGTACAGTTTGAG
pre-let7g-QRT-REV	GCAAGGCAGTGGCCTGTACAGTTATC
U6-QRT-FWD	CGCTTCGGCAGCACATATAC
U6-QRT-REV	AGGGGCCATGCTAATCTTCT
GAPDH-QRT-FWD	CTCAAGGGCATCCTGGGCTA
GAPDH-QRT-REV	ATGAGGTCCACCACCCTGTT

Lin28A-mRNA-FWD	AAGCGCAGATCAAAAGGAGA
Lin28A-mRNA-REV	CTGATGCTCTGGCAGAAGTG
